# Multiscale Engineering of PEO Electrolytes for High‐Voltage and Ultrastable Solid‐State Lithium Batteries With Exceptional Room‐Temperature Performance

**DOI:** 10.1002/anie.202523382

**Published:** 2026-02-18

**Authors:** Xuefan Liu, Bowen Zhang, Congcong Zhang, Xueyu Zhou, Xu Liu, Teng Liu, Shifeng Hou, Qifeng Zheng, Lu Wang, Linglong Kong, Shanqing Zhang

**Affiliations:** ^1^ Key Laboratory of Low‐Carbon and Green Agriculture Chemistry in Universities of Shandong College of Chemistry and Material Science Shandong Agricultural University Tai'an Shandong China; ^2^ State Forestry and Grassland Administration Key Laboratory of Silviculture in Downstream Areas of the Yellow River College of Forestry Shandong Agricultural University Tai'an Shandong China; ^3^ Institute For Sustainable Transformation School of Chemical Engineering and Light Industry Guangdong University of Technology Guangzhou Guangdong China

**Keywords:** anion coordination effects, high‐voltage cathode, multiscale engineering, PEO electrolytes, room‐temperature operation

## Abstract

Poly(ethylene oxide) (PEO) electrolytes show significant promise for flexible solid‐state batteries, yet face insufficient ion transport kinetics and interfacial stability. Herein, we propose a multiscale engineering that synergistically modulates both macro‐mesoscopic polymer architectures and microscopic solvation configurations by incorporating poly(ethylene oxide)‐poly(2‐dimethylaminoethyl methacrylate) nitrate (PEG‐PDMAEMAH^+^·NO_3_^−^) additives. The introduced polycationic chains effectively disrupt PEO crystallinity, promote segmental motion and enhance the solubility of NO_3_^−^. Importantly, NO_3_^−^ with high donor number can competitively coordinate with Li^+^, weakening the ethylene oxide‐Li^+^ chelation and thereby boosting bulk Li^+^ mobility. The resulting anion‐rich solvation structure lowers the desolvation energy barrier and fosters the formation of robust, highly conductive inorganic‐rich solid electrolyte interlayers at both the cathode and anode, which enhances the interfacial kinetics and high‐voltage tolerance. Consequently, the engineered PEO electrolyte enables lithium metal batteries to operate stably at near‐room temperature (30°C) without liquid plasticizers. Correspondingly, the 4.3 V LiNi_0.8_Co_0.1_Mn_0.1_O_2_ cell achieves stable cycling over 500 cycles at 0.2 C with a high capacity retention (82.7%), while the LiFePO_4_ cell maintains stable operation for 1200 cycles at 0.5 C (30°C). The proposed strategy creates an avenue to accelerate the eventual commercialization of the polymer electrolytes for solid‐state lithium batteries.

## Graphical abstract

Incorporating poly(ethylene oxide)‐poly(2‐dimethylaminoethyl methacrylate) nitrate (PEG‐PDMAEMAH^+^·NO_3_^−^) enhances amorphous‐phase conduction with optimized solvation in engineered PEO–PD–Li electrolytes for solid‐state lithium metal batteries.

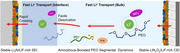

## 1 Introduction

Compared with traditional liquid electrolytes, solid‐state electrolytes exhibit superior mechanical strength, which could enable compact battery architectures and eliminate the risks of electrolyte leakage, thereby synergistically enhancing both safety and energy density [1, 2]. Among these, solid polymer electrolytes (SPE) have garnered significant attention for their advantages of high flexibility and interfacial compatibility with electrode materials, as well as their unique capability on simpler packaging, versatile geometric designs, and scalable production [3]. Poly(ethylene oxide) (PEO)‐based electrolytes, recognized as the most extensively studied SPE system, have achieved commercial implementation in electric vehicles by the Bolloré Group [4, 5]. This prominence stems from the elevated donor number (DN) of ethylene oxide (EO) units and the intrinsic chain flexibility, which collectively enable superior lithium salt solvation capacity and ion transport performance compared to conventional polymer matrixes. Nevertheless, the electrochemical performance of PEO electrolytes remains insufficient to meet the requirements of advanced lithium battery applications, especially under high‐voltage operating conditions at room‐temperature.

**FIGURE 1:**
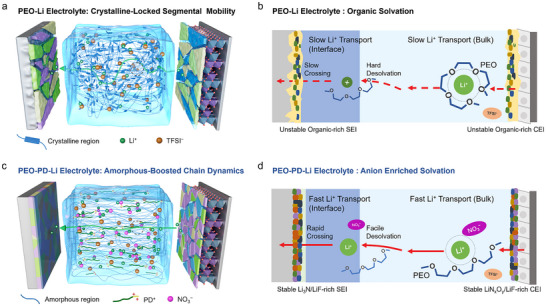
Schematic illustration of a multiscale engineering strategy for the simultaneous regulation of PEO crystallinity and Li^+^ solvation structure in solid‐state lithium batteries. In the pristine PEO electrolyte (PEO–Li), Li^+^ transport is impeded by (a) polymer crystallinity restricting segmental motion and (b) strong EO–Li^+^ chelation. This chelation also leads to an organic‐dominated solvation structure, resulting in unstable interfacial layers. In the engineered PEO electrolyte (PEO–PD–Li), Li^+^ transport is enhanced through (c) amorphization‐boosted segmental motions and (d) reduced EO‐Li^+^ chelation due to the competitive coordination from NO_3_^−^ anions. The resultant anion‐rich solvation structure promotes the formation of stable SEI on the lithium anode and stable CEI on the high‐voltage NCM811 cathode.

PEO electrolytes show certain similarities with liquid electrolytes on the solvation structure, with each Li^+^ being coordinated by five or six oxygen atoms from EO units. The transport of Li^+^ between the cathode and anode also involves three steps: (a) solvated Li^+^ diffusion through the bulk electrolyte, (b) Li^+^ desolvation before crossing the solid electrolyte interphase (SEI/CEI), and (c) naked Li^+^ migration across the SEI/CEI layer [6, 7]. The overall electrochemical kinetics is governed by synergistic optimization of this sequential triad. While high‐molecular‐weight PEO (e.g., 600 000 g mol^−1^) improves mechanical stability, it sacrifices ion transport kinetics compared to liquid electrolytes, exhibiting disparities in both solvation environment and transport dynamics (Figure 1a,b).

Microstructurally, the entangled PEO chains create a rigid solvation environment due to the strong chelation effects from high‐DN EO oxygen atoms, differing from the labile solvation structures in liquid electrolytes. This rigid chelation architecture impedes the disruption of the organic solvation layer, thereby diminishing the desolvation kinetics and Li^+^ transference number (*t*_Li+_), and fostering organic‐rich interphases with poor ionic conductivity. (Figure 1b). Mesoscopically, Li^+^ migration in PEO relies on segmental chain motion for ion hopping between coordination sites, which often leads to a reduction in ion conduction speed as compared with the fast “vehicular” conduction model in liquid electrolytes [8]. Macroscopically, since ion transport in PEO predominantly occurs in amorphous regions, its high crystallinity significantly impedes Li^+^ mobility at room temperature (Figure 1a) [9].

Therefore, the multiscale regulation could improve the performance of PEO electrolytes toward commercial applications [10]. Extensive efforts have focused on crystallinity suppression and lithium salt dissociation enhancement through modifying polymer chain [11, 12, 13], adding plasticizers [14, 15], and incorporating organic/inorganic fillers [16, 17]. Particularly, achieving room‐temperature operation in PEO systems often necessitates liquid plasticizers [18, 19, 20], which inevitably induces the solvation of the polymer and the formation of a gel, paradoxically sacrificing intrinsic safety advantages.

As to the solvation structure, weak‐coordination polymer chains or Li^+^ transport mediators are often introduced to create accelerated ion‐conduction pathways [13, 21, 22]. Lewis‐acids were also applied to improve *t*_Li+_ by anchoring TFSI^−^ anions [23, 24]. Nevertheless, anion coordination chemistry, which has been well established as an effective approach for regulating solvation structures and optimizing Li^+^ transport in liquid electrolyte systems, remains underutilized in solid polymer electrolytes [25, 26, 27, 28, 29]. In this regulation strategy, the high‐DN anions with the optimal additive amount could restructure the solvation sheath to weaken organic solvent‐Li^+^ binding due to their strong Li^+^ coordination, and achieve raised ion conduction in bulk electrolyte. In addition, the anion enriched solvation structure would facilitate the formation of robust SEI/CEI layers, and improve desolvation kinetics [30]. Therefore, the anion coordination strategy demonstrates potential in simultaneously enhancing ionic conductivity and interfacial properties to boost voltage tolerance of PEO electrolytes, which warrants systematic investigation. However, the limited solubility of the high‐DN anions needs to be carefully considered to achieve effective regulation of Li^+^ transport dynamics.

**SCHEME 1:**
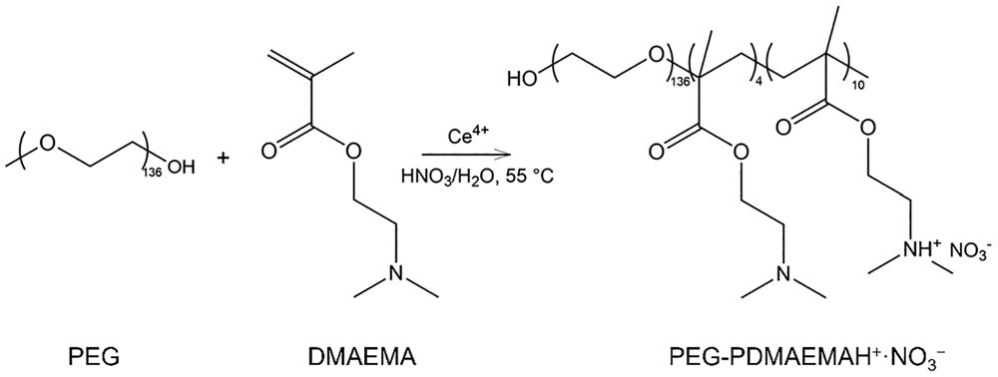
Synthesis of PEG‐PDMAEMAH^+^·NO_3_^−^ copolymer.

Herein, we propose a simple approach to coordinatively optimizing macroscopic polymer architectures, mesoscopic segmental motion dynamics and microscopic solvation structures of the PEO electrolyte, demonstrating proof‐of‐concept multiscale electrolyte engineering to achieve high‐performance PEO electrolytes (Figure 1c,d). The anion effect on Li^+^ transport and interfacial properties has also been extensively studied and discussed. Specifically, poly(ethylene glycol)‐poly(2‐dimethylaminoethyl methacrylate) nitrate (PEG‐PDMAEMAH^+^·NO_3_^−^, denoted as PD) copolymers were synthesized via one‐step free radical copolymerization (Scheme 1), and further introduced into the high‐molecular‐weight PEO (*M*v ≈ 600 000 g mol^−1^) electrolyte with a 20 wt% addition to obtain the PEO‐PD‐Li electrolyte.

The PD copolymer could easily dissociate into PEG‐PDMAEMAH^+^ (PD^+^) polycations and NO_3_^−^ anions, where the bulky and flexible PD^+^ polycations enable exceptional NO_3_^−^ solubility, surpassing conventional alkali nitrate systems [31, 32]. Mechanistically, the short‐chain PD^+^, which exhibits good compatibility with PEO, could effectively promote the amorphization and segmental motion of PEO (Figure 1c). Also, it could provide another Li^+^ conduction path through the PEG segments, avoiding the phase incompatibility issues or ion‐blocking interfaces commonly observed in conventional non‐conductive plasticizers or inorganic fillers [22, 33].

More importantly, the strong electron‐donating capability of high‐DN NO_3_^−^ anions (22.2 kcal mol^−1^) enables their preferential coordination with Li^+^ ions in the primary solvation shell [34, 35]. This coordination effectively enhances Li^+^ transport kinetics by weakening the strong chelation effect induced by EO units. Concurrently, the high‐DN NO_3_^−^ anion enriched solvation structures facilitate the lowering of desolvation energy barrier, and promotes the formation of the robust and high conductive Li_3_N/LiF rich SEI and LiN_x_O_y_/LiF rich CEI, which could ensure exceptional cycling stability and high‐voltage cathode compatibility (Figure 1d) [36, 37]. Thus, the addition of PD could achieve accelerated Li^+^ transport kinetics both in the bulk electrolyte and interphases. The as‐engineered electrolyte enables superior room‐temperature performance of both high‐voltage LiNi_0.8_Co_0.1_Mn_0.1_O_2_ (NCM811) and LiFePO_4_ (LFP) cathodes without the need for any liquid additives. The corresponding Li||LFP cell could stably operate 1200 cycles at 0.5 C with a low decay rate of 0.0064% per cycle at 30°C. And the Li||NCM811 cell shows excellent stability with a high retention rate of 82.7% at 0.2 C over 500 cycles (30°C).

## 2 Results and Discussion

### 2.1 Polymer and Solvation Structure Regulation

The designed PD block copolymers as electrolyte regulator are synthesized via an optimized one‐step free radical polymerization adapted from the previous literature [38]. Specifically, poly(ethylene glycol) (PEG, M_n_ ≈ 6000 g mol^−1^) first reacts with cerium (IV) in water to generate PEG radicals, and initiates the copolymerization with 2‐(dimethylamino)ethyl methacrylate (DMAEMA) (Figures S1,S2). In this process, the tertiary amine (R_3_N) of DMAEMA binds with a proton (H^+^) from nitric acid through the lone pair electrons, forming a quaternary ammonium salt (PEG‐PDMAEMAH^+^·NO_3_^−^, PD, Scheme 1). It could be found the as‐prepared PD are in solid state (Figure S3). Successful copolymerization is confirmed by a series of characterizations (Figures S4–S8). As observed in the Fourier‐transform infrared spectroscopy (FTIR) of PD (Figure S4), the characteristic carbonyl (C═O) stretching vibration emerges at 1730 cm^−1^, accompanied by the disappearance of the C═C stretching band at 1645 cm^−1^. While in ^1^H nuclear magnetic resonance (NMR) spectroscopy (Figure S5), the proton resonances at 3.5–3.8 ppm correspond to ether group protons, while signals at 0.8–1.0 and 2.9 ppm are assigned to methyl protons in the DMAEMA chains and methyl groups bonded to nitrogen atoms, respectively. Notably, the absence of characteristic vinyl proton signals (∼6.5 ppm) confirms complete copolymerization. X‐ray photoelectron spectroscopy (XPS) analysis further corroborates the molecular structure. The N 1s spectrum (Figure S6) reveals two distinct peaks that assigned to (CH_3_)_2_NH^+^‐ (402.2 eV) and the nitrate counterions (NO_3_^−^, 406.7 eV) [39]. The C 1s spectrum (Figure S7) displays a characteristic peak at 288.90 eV corresponding to the carboxylate group (‐COO^−^) derived from DMAEMA monomer [40]. The C 1s and N 1s XPS spectra were jointly used to determine the molecular formula of PD shown in Scheme 1. The lithium salt concentration in the SPE was fixed at an EO:Li ratio of 11:1, as determined by the lowest glass transition temperature (*T*_g_) in differential scanning calorimetry (DSC) analyses (Figure S9) and high ionic conductivity (Figure S10). The PD additive content was then optimized by evaluating PEO electrolytes with 10, 15, 20, and 30 wt% PD in Li||LiFePO_4_ cells at 30°C. The composition with 20 wt% PD exhibits the highest specific capacity and cycling stability (Figure S11), and was therefore selected for all subsequent studies. Conventional PEO electrolyte (PEO‐Li) and PEG (M_n_ ≈ 8000 g mol^−1^, similar to PD) modified PEO electrolyte (PEO–PEG–Li) were also prepared as comparative materials to illustrate the functions of PD in the polymer and solvation structure, bulk conductivity and interfacial properties.

**FIGURE 2:**
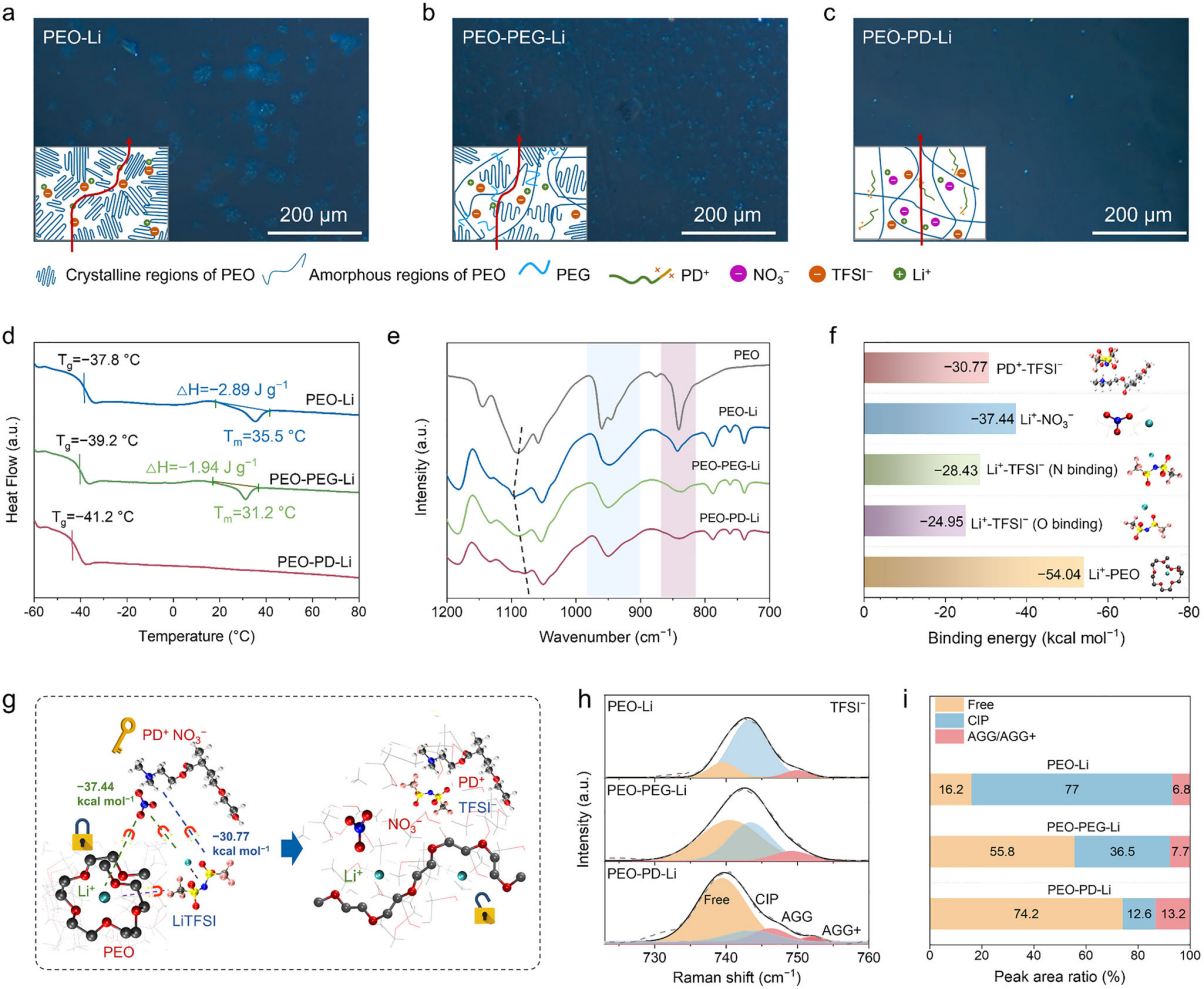
The macro‐mesoscopic regulatory role of PD on the crystallinity and segmental dynamics of PEO. (a–c) POM images for different electrolytes. The insets are the conceptual diagrams showing Li^+^ migration routes, where red arrows indicate conduction pathways in each electrolyte. (d) DSC plots and (e) FTIR spectra for the electrolytes. (f) Binding energies drawn from the density functional theory (DFT) calculations. (g) Mechanism illustration of PD^+^ and NO_3_^−^ in promoting the lithium salts dissociation and releasing Li^+^ from the chelation of EO units. (h) Raman spectra and (i) the corresponding peak area ratio of various TFSI^−^ clusters of the electrolytes.

The regulatory role of PD was systematically investigated across multiple scales. First, PD's influence on PEO crystallinity reduction and lithium salt dissociation behavior was evaluated. As a linear polymer with a regular molecular structure, PEO exhibits strong crystallization tendencies as manifested by the abundant spherulites domains observed in polarized optical microscopy (POM) images (Figure S12). Although the lithium salts could function as the plasticizer to partially reduce crystallinity [41], the residual observable crystalline regions still restrict the mobility of Li^+^ (Figure 2a). In addition, excessive lithium salt loading shows limited efficacy in further crystallization suppression, and would risk deteriorating ionic conductivity due to the steric effect of the undissolved salt (Figures S9, S10) [42]. While the incorporation of short‐chain PEG could disrupt the crystallinity of PEO with the reduced spherulite size, the inhibition was still incomplete (Figure 2b). Remarkably, PD could almost eliminate the crystalline regions of PEO, possibly attributed to its comb‐shaped molecular architecture with the compatible PEG short chains (Figure 2c). DSC curves reveal a decreased *T*_g_ (−41.2°C) and complete absence of melting peak for PEO–PD–Li electrolyte, indicating its amorphous nature, which could also be ascertained by the reduced and broadened FTIR peaks at 1467 and 2876 cm^−1^ (Figures 2d, S13) and the vanished characteristic diffraction peaks in x‐ray diffraction (XRD, Figure S14) [21]. The disruption of the crystalline enhances the mobility of PEO chains and blazes more routes for the rapid Li^+^ transportation. In addition, with containing the PEG segments, PD exhibits excellent compatibility with PEO that facilitates molecular‐level integration and electrolyte homogeneity (Figure S15). This structural configuration concurrently creates an additional Li^+^ transport pathway and ensures the continuous ion conduction, overcoming the poor interfacial compatibility of inorganic fillers [22] and discontinuous ion transport in non‐conductive organic fillers [33].

The mesoscopic conformational dynamics of polymer chains were probed through specific FTIR vibrations. The FTIR peaks at 844 and 950 cm^−1^ correspond to the CH_2_ rocking and bending vibration, respectively [10, 43], which are sensitive to the conformational changes, and their broadening after PD addition indicates the increased torsional angle and conformational diversity, demonstrating the mobility of PEO chains is increased (Figure 2e). Meanwhile, the C─O─C stretching vibration of PEO at 1090 cm^−1^ shifts to ∼1097 cm^−1^ with the formation of Li^+^–EO complexation in PEO–Li electrolyte, and a partial reversion is observed due to reduced complexation after PD incorporation, demonstrating the reduced Li^+^–EO coordination (Figure 2e) [44]. Moreover, molecular dynamics (MD) simulations (Figure S16, Table S1) demonstrate the introduced PD induces an enlarged free volume in the polymer matrix, thereby enhancing Li^+^ hopping along the polymer segments (Figure S17), Therefore, the incorporation of PD significantly enhances the segmental mobility of PEO, and simultaneously reduces the strong Li^+^ coordination, thereby facilitating rapid Li^+^ transport in bulk electrolyte.

As the amorphous regions increased, the enhanced interaction between PEO chains and lithium salts could motivate the dissociation of lithium salts. More importantly, the zwitterionic PD exhibits strong electrostatic interactions with lithium salts, with binding energies of −30.77 kcal mol^−1^ for PD^+^ and TFSI^−^, and −37.44 kcal mol^−1^ for NO_3_^−^ and Li^+^ (Figure 2f). This synergistic effect promotes Li‐TFSI dissociation (−28.43 kcal mol^−1^), resulting in 74.2% free TFSI^−^ content in the PEO–PD–Li system, far exceeding that in PEO–Li (16.2%) and PEO–PEG–Li (55.8%), as derived from the Raman spectroscopy (Figure 2g–i). Additionally, the observed red shifts in the FTIR and XPS spectra for the ─SO_2_ and ─CF_3_ peaks of TFSI^−^ in PEO–PD–Li are indicative of the reduced coordination strength with Li^+^, corroborating the enhanced salt dissociation (Figures S18, S19). Concurrently, XPS peak variations for amine groups confirm the dissociation of PD^+^ from the strongly electron‐withdrawing NO_3_^−^ and the subsequent binding with TFSI^−^ (Figure S20). Therefore, the incorporated PD could create expanded amorphous areas, improved segmental dynamics, higher free Li^+^ concentration and continuous pathways for accelerating Li^+^ conduction at the macro‐mesoscale. Following the generation of abundant free Li^+^ ions, loosening the tight EO–Li^+^ coordination constitutes the second crucial step toward achieving fast ion transport in electrolytes [10]. Herein, high‐DN NO_3_^−^ of PD could effectively coordinate with Li^+^ ions, and loosen the tight Li^+^–EO coordination to create a more favorable micro structure in PEO‐based electrolytes (Figure 2g).

**FIGURE 3:**
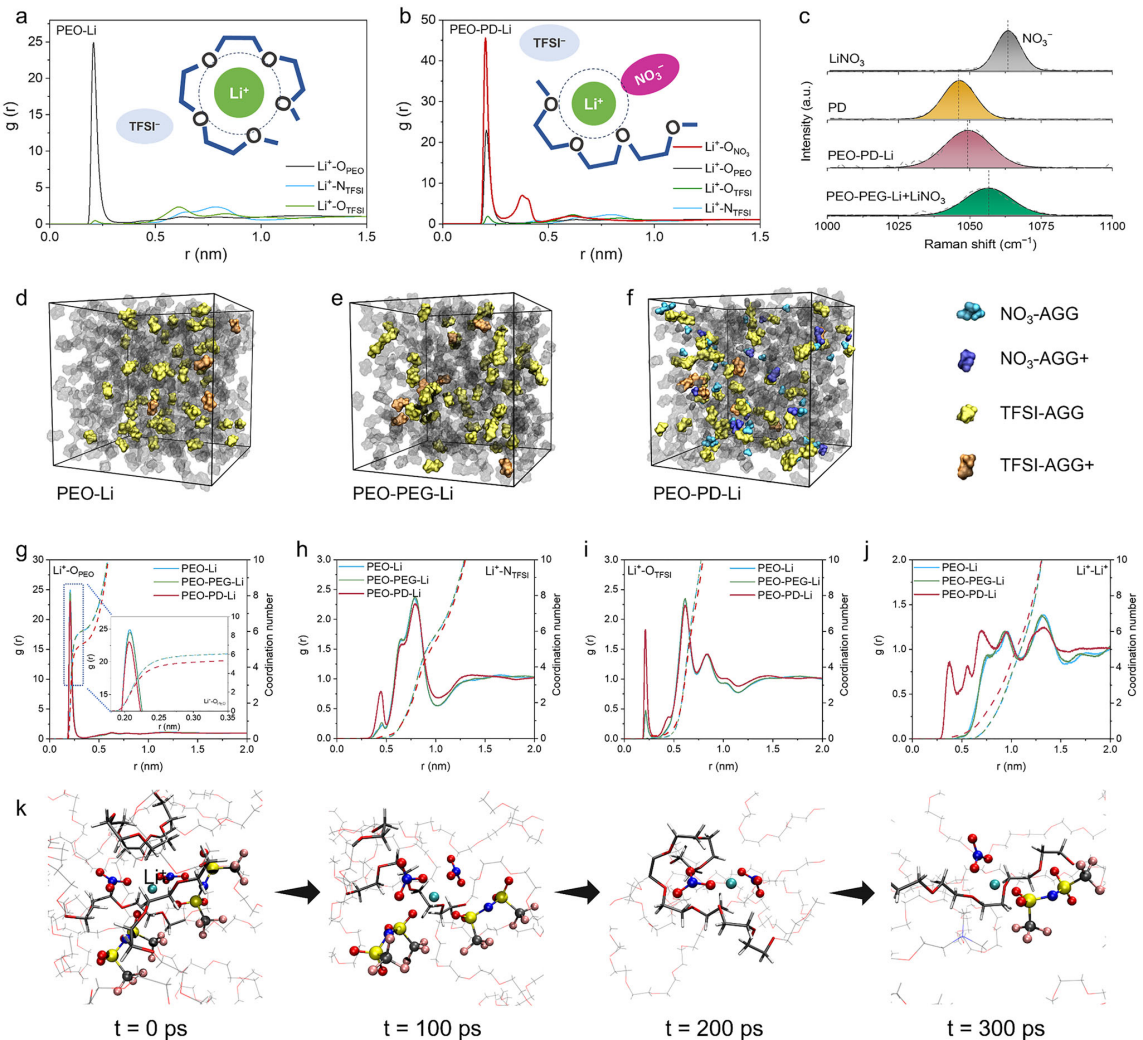
The microscopic regulatory role of PD on the solvation structure via the anion effect. The Li^+^ solvation structure calculated from MD simulation for the (a) PEO–Li and (b) PEO–PD–Li electrolytes. Schematic diagrams are shown in the insets. (c) Raman spectra of LiNO_3_ powders, PD powders, PEO–PD–Li and PEO–PEG–Li+LiNO_3_ electrolyte. Snapshots of the MD simulations demonstrate the correlated solvation configurations, for (d) PEO–Li, (e) PEO–PEG–Li, and (f) PEO–PD–Li electrolyte. The blue and dark blue represent the AGG and AGG+ structures for NO_3_^−^, while the yellow and brown represent the AGG and AGG+ structures for TFSI^−^. The RDF (solid line) and the corresponding coordination number plots (broken lines) of (g) Li^+^─O_PEO_, (h) Li^+^─N_TFSI_, (i) Li^+^─O_TFSI_, and (j) Li^+^─Li^+^ pairs calculated from MD simulation for the electrolytes. (k) Screenshots of Li^+^ trajectory in PEO–PD–Li electrolyte by MD simulation under the electric field.

MD simulations are conducted to provide insights for this optimized solvation structure. In PEO–Li and PEO–PEG–Li systems, radial distribution function (RDF) analyses indicate that the coordination of Li^+^ is dominated by the oxygen atoms in PEO–Li system (∼0.2 nm, Figures 3a, S21). In the PEO–PD–Li electrolyte, the high‐DN NO_3_^−^ anions extensively participate in the inner Li^+^ solvation sheath alongside PEO oxygen atoms, due to their high affinity with Li^+^ (Figure 3b). This structural evolution is further verified by Raman spectroscopy (Figure 3c). When PD is introduced into the PEO electrolyte, the NO_3_^−^ peak undergoes a blue shift from 1046.2 to 1049.5 cm^−1^, corresponding to its dissociation from the weak Lewis acid PD^+^ and subsequent combination with the relatively strong Lewis acid Li^+^. Importantly, this effect cannot be achieved by simple LiNO_3_ addition due to its limited solubility, which inhibits the anion from participating in solvation structure reorganization. When LiNO_3_ is added into the PEO–PEG–Li electrolyte (marked as PEO–PEG–Li+LiNO_3_), the NO_3_^−^ vibration peak only shifts to 1057.3 cm^−1^, which is closer to that of pristine LiNO_3_ (1063.5 cm^−1^), indicating its limited dissociation. This insufficient anion activation directly leads to the negligible enhancement in the electrochemical performance (Figure S22).

More importantly, we found that the introduction of PD could significantly increase the number of anion–cation aggregates (AGG, Figure 3d–f). On the one hand, the strongly coordinating NO_3_^−^ tends to form ionic aggregates such as Li_2_(NO_3_^−^)^+^ (NO_3_‐AGG) and Li_3_(NO_3_^−^)^2+^ (NO_3_‐AGG+), as shown in the snapshots of PEO–PD–Li (Figure 3f). On the other hand, NO_3_^−^ could liberate Li^+^ from the PEO chain confinement and enhance the chance of Li^+^–TFSI^−^ contact [45], as confirmed by reduced coordination number of Li─O_PEO_ (Figure 3g) alongside an increased presence of TFSI^−^ in the primary solvation shell of Li^+^ in the RDF analysis (Figure 3h,i). Experimentally, the Raman spectra in Figure 2h verify the increase in the proportion of TFSI–AGG. Thus, the total quantity of the ionic aggregates in PEO–PD–Li electrolyte shows a notable increase, in consistence with the RDF of Li^+^─Li^+^ pairs (Figure 3j). Under the electric field, the positively charged AGG structures could accumulate at the negatively charged Li surface, leading to accelerated anion decomposition to form an anion‐derived inorganic‐rich SEI [46], as confirmed by experiments discussed in later sections. Figure 3k shows the screenshots of Li^+^ trajectory in PEO–PD–Li by MD simulation under the electric field. It can be observed that the PEO chain is independent and flexible, meaning the entanglement of PEO chains and their chelation effect on Li^+^ is disrupted by PD, which contributes to the as‐observed anion‐enriched solvation structure in Figure 3k.

Therefore, the PD^+^ polycations could effectively enhance the mobility of PEO chain and lithium salts dissolution, while the high‐donor anions could moderate the over strong association between Li^+^ and PEO chains, thus fast Li^+^ transport in the bulk electrolyte is achieved. In addition, the anion enriched Li^+^ solvation structure in PEO–PD–Li electrolyte with the advantageous AGG structures would help to improve the interfacial properties as illustrated in the later section.

### 2.2 Bulk Properties of the Electrolytes

**FIGURE 4:**
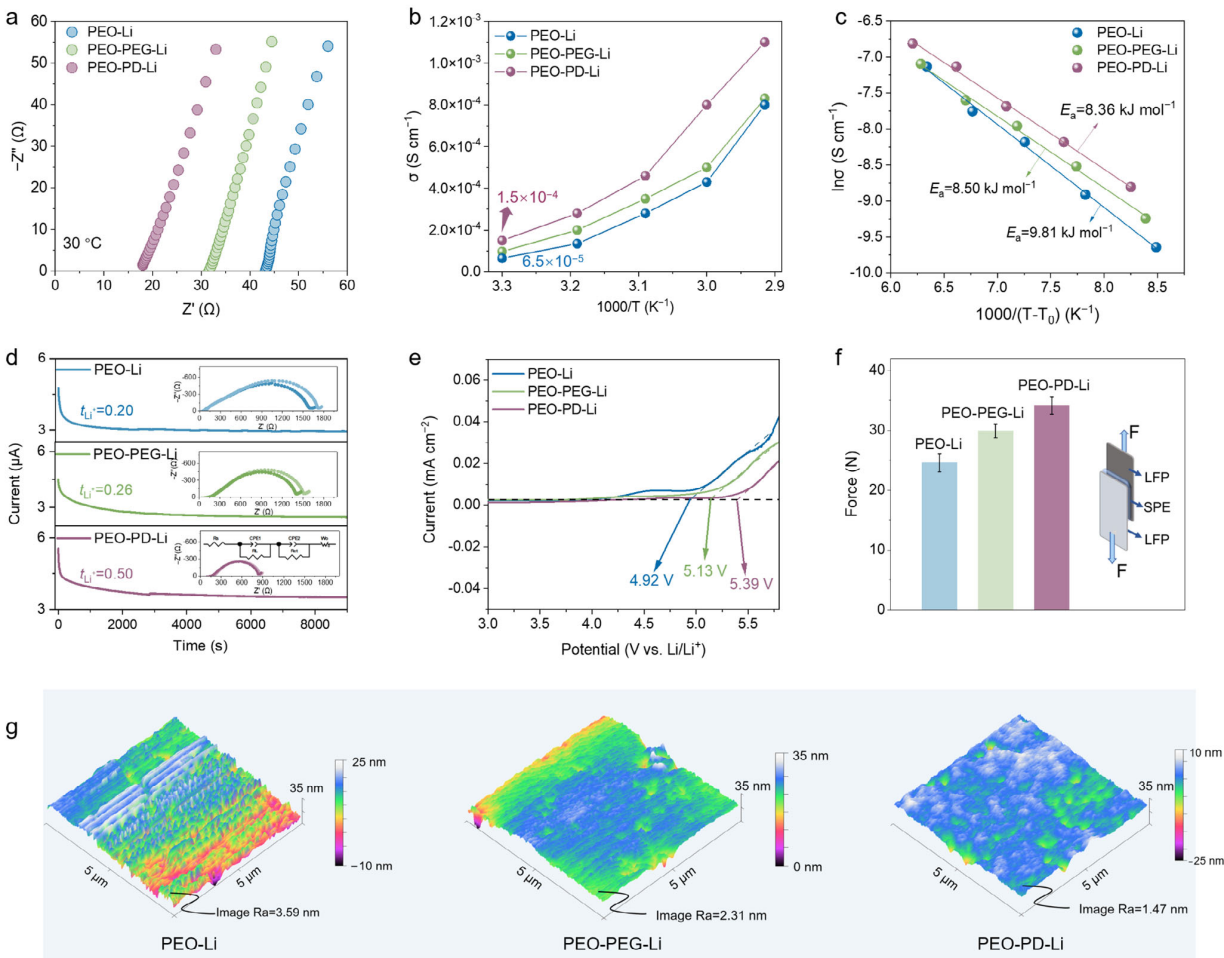
Bulk properties of the electrolytes. (a) EIS at 30°C, (b) ionic conductivity at different temperatures, and (c) the conductivity‐temperature data in the Vogel–Tamman–Fulcher coordinates. The *T*_0_ values labeled in the graph are taken from the DSC experiments (*T*_g_−50). (d) Current‐time plots and EIS test of Li||Li symmetric cells for deriving *t*_Li+_ for these three electrolytes. (e) LSV plots for the SS||SS cells assembled with different electrolytes. (f) Adhesion force test results (error bars indicate mean±standard deviation of three independent measurements) and (g) AFM images for the prepared electrolytes.

To systematically evaluate the enhanced ion transport properties in the bulk electrolytes conferred by the multiscale electrolyte engineering, systematic characterizations were performed. First, electrochemical impedance spectroscopy (EIS) of stainless‐steel symmetric cells (SS||SS) demonstrates a significantly reduced impedance across temperature ranges, reflecting the enhanced ionic conductivity (Figures 4a,b and S23). Particularly, the PEO–PD–Li electrolyte achieves an ionic conductivity of 1.5×10^−4^ S cm^−1^ at 30°C without adding any liquid, much higher than that of PEO–Li (6.5×10^−5^ S cm^−1^), endowing the solid batteries with significant potential for reliable operation at near‐ambient temperature. Consistent with this improvement, the VTF activation energy (*E*_a_) is reduced to 8.36 kJ mol^−1^ for PEO–PD–Li, compared to 9.81 kJ mol^−1^ for PEO–Li, as shown in Figure 4c.

The related *t*_Li+_ is an important indicator of Li^+^ transport ability, which is determined by Bruce–Vincent method based on current‐time plots of Li||Li symmetric cells (Figure 4d). The *t*_Li+_ of the PEO–PD–Li electrolyte could be raised to 0.5, nearly double values of PEO–Li (*t*_Li+_ = 0.2) and PEO–PEG–Li (*t*_Li+_ = 0.26). This elevated *t*_Li+_ arises from NO_3_^−^‐induced loosened Li^+^ coordination structures for fast transport and PD^+^‐mediated TFSI^−^ immobilization. Concurrently, the optimized electrolyte exhibits an expanded electrochemical stability window (5.39 V vs. Li/Li^+^) relative to PEO–PEG–Li (5.13 V vs. Li/Li^+^) and PEO–Li (4.92 V vs. Li/Li^+^), enables possible compatibility with high‐voltage cathodes (Figure 4e). To strictly define the feasible electrochemical window, electrochemical floating tests were conducted. The results reveal that whereas the PEO–Li electrolyte suffers significant oxidative currents above 4.2 V, the PEO–PD–Li electrolyte remains stable against oxidation up to 4.6 V with minimal leakage current (< 20 µA), validating its contribution to the stable cycling performance of NCM811 cells at high voltages (Figure S24). This improvement stems from anion‐reinforced solvation structures that effectively passivate electrode surfaces, as detailed in the subsequent analysis. Furthermore, PD modification also endows the fabricated SPE films with superior electrode adhesion (Figure 4f) and exceptional surface homogeneity (Figure 4g), ensuring interfacial wettability and compatibility during ambient‐temperature operation. Therefore, it can be concluded that the Li^+^ transfer kinetics in the bulk electrolyte is elevated.

### 2.3 Interfacial Properties

**FIGURE 5:**
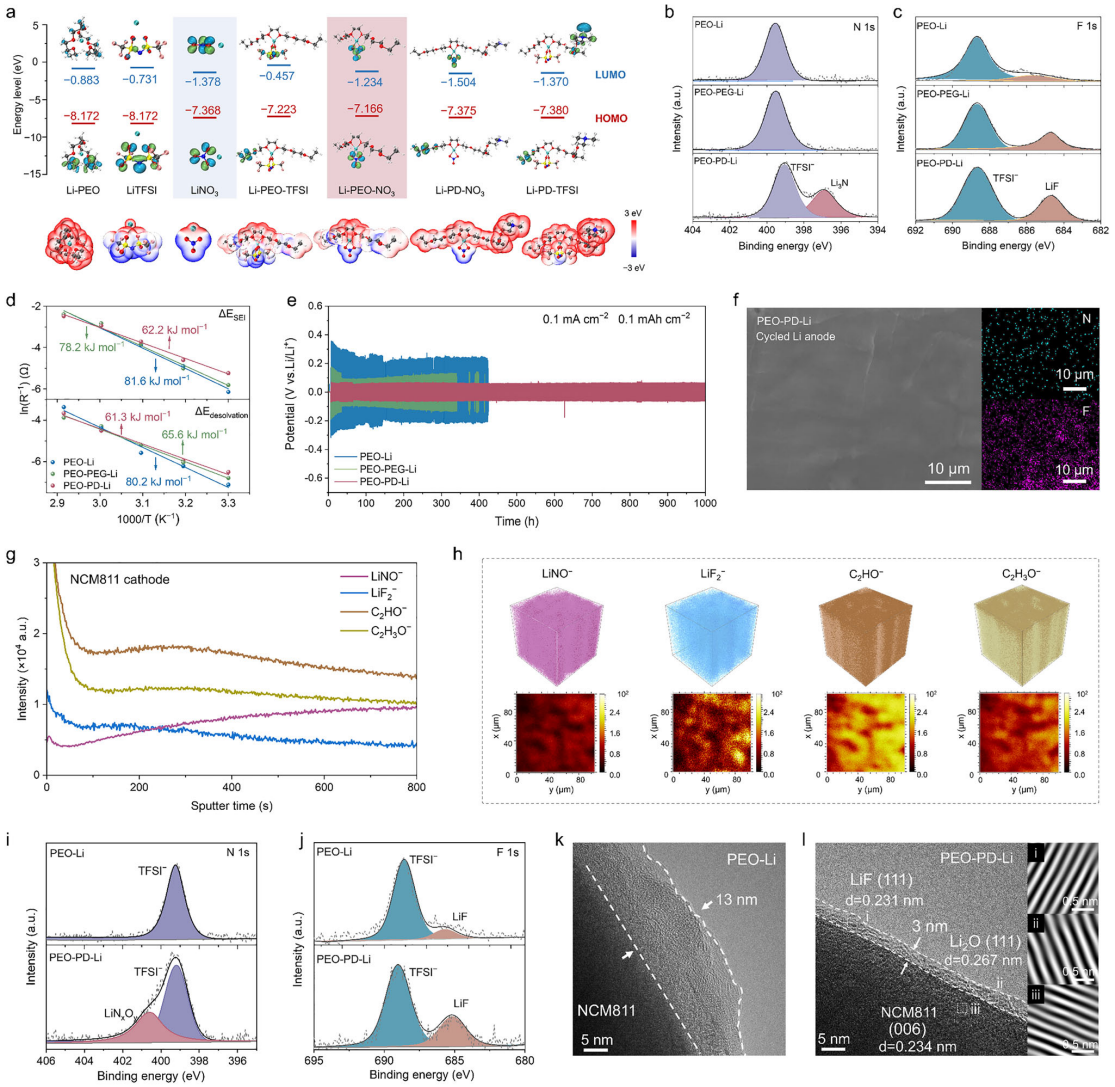
Interfacial properties at the anode and cathode. (a) Calculated HOMO and LUMO energy levels and the corresponding electrostatic potential distribution of the typical coordination structures. (b) N 1s and (c) F 1s XPS spectra of the cycled Li anode in the cells with different electrolytes. (d) Arrhenius plots of the *R*_SEI_ and *R*_desolvation_ in Li||Li symmetric cells to drawn the corresponding Δ*E*_a_. (e) The electrochemical performance of Li||Li symmetrical cells assembled with different electrolytes. (f) The SEM and EDS mapping images of the lithium anode after cycling in the Li||Li symmetric cell with PEO–PD–Li electrolyte. (g) TOF‐SIMS depth profiles and (h) 3D tomography with 2D color mappings of LiNO^−^, LiF_2_^−^, C_2_HO^−^, and C_2_H_3_O^−^ in the derived CEIs of NCM811 cathode after 50 cycles in the cell assembled with PEO–PD–Li electrolytes. (i) N 1s and (j) F 1s XPS spectra of the NCM811 cathodes after 50 cycles. HRTEM images of the NCM811 particles after 50 cycles in the (k) PEO–Li and (l) PEO–PD–Li based cells, with the corresponding inverse fast Fourier transform patterns shown for the selected area in (l).

Except for improving the bulk kinetics of the electrolyte, the interfacial properties, including Li^+^ desolvation at the electrode–electrolyte interface and migration through the interfacial layer (SEI/CEI), are also vital for guaranteeing the high ion transport efficiency across the entire system (Figure 1). These interfacial properties are closely correlated with the primary solvation shell of Li^+^. The lowest unoccupied molecular orbital (LUMO) energy levels of the dominant solvation configurations extracted from the MD simulation were first calculated by DFT. Li–NO_3_ and Li–PEO–NO_3_ exhibit lower LUMO energy levels than that of Li–PEO, Li–TFSI and Li–PEO–TFSI, facilitating their preferential reductive decomposition on Li anode to create a Li_3_N‐rich SEI (Figure 5a,b). Furthermore, the positively charged NO_3_–AGG and TFSI–AGG clusters are more easily accumulated at the negatively charged Li surface, accelerating the decomposition of the carried anions to form an anion‐derived inorganic‐rich SEI, as verified by the pronounced Li_3_N and LiF signals in the XPS spectra of the cycled Li anode (Figure 5b,c). As Li_3_N possesses superior lithium affinity, high ionic conductivity and unique thermodynamic stability [36, 47], the relevant energy barriers of Li^+^ transport through the SEI (Δ*E*_SEI_) could be significantly reduced (Figures 5d, S25, and Table S2).

Electrostatic potential (ESP) mapping discloses that negative charges are concentrated on the anions in these dominant solvation configurations (Figure 5a), which bear stronger electric field repulsion compared to neutral solvent molecules and are easier to separate with Li^+^ on the electrode surface (Figure S26) [48]. Therefore, the anion‐enriched solvation structures are more prone to desolvate, supported by the fact that PEO–PD–Li electrolyte exhibits the lowest desolvation energy barrier (Δ*E*_desolvation_, 61.3 kJ mol^−1^, Figures 5d, S25 and Table S1)[49, 50]. Furthermore, the PEO–PD–Li electrolyte shows the lowest Li^+^ transport barriers through the electrode–electrolyte interface (Δ*E*_SEI_) with the highest exchange current density (1.6 × 10^−2^ mA cm^−2^_‐PEO–PD–Li_ vs. 1.1 × 10^−2^ mA cm^−2^_‐PEO–PEG–Li_ and 0.7×10^−2^ mA cm^−2^_‐PEO–Li_, Figure S27). In contrast, the Li^+^ solvation shell of PEO–Li electrolyte is tightly chelated by PEO chains, leading to tough desolvation and the formation of organic SEI layer with low ionic conductivity, consequently induces high Δ*E*_desolvation_, high Δ*E*_SEI_, and low exchange current density (Figures 5d, and S25–S27). The improved interfacial kinetics and the robust SEI layer resulting from the PEO–PD–Li electrolyte could stabilize Li anode with excellent cycle performance. The Li||Li symmetric cell assembled with PEO–PD–Li electrolyte could stably operate over 1000 h with small polarization at 0.1 mA cm^−2^ (Figure 5e), and perform well at varying currents from 0.1 to 2.6 mA cm^−2^ (Figure S28). In addition, after the repeated dissolution/deposition processes for 100 cycles, the Li anode in Li|PEO–PD–Li|Li cell still maintains a flat and smooth surface with uniformly distributed N and F elements (Figure 5f). In contrast, the Li anodes from the cells using PEO–Li and PEO–PEG–Li electrolytes show rugged morphology (Figure S29). In addition, as observed in the optical microscope, the Li surface in the Li|PEO–PD–Li|Li cell remains flat throughout the deposition, while that of Li|PEO–Li|Li cell occurs obvious fluctuation (Figure S30). The uniform Li plating/stripping behavior motivated by PEO–PD–Li electrolyte would enhance the availability and safety for fabricating high energy density lithium metal batteries.

On the cathode side, the surface passivation by CEI is crucial for the stability of high‐voltage cathode materials (e.g., NCM811). DFT calculations show that the highest occupied molecular orbital (HOMO) energy levels of Li–PEO (−8.172 eV) significantly increase after complexing with NO_3_^−^ (−7.166 eV for Li–PEO–NO_3_, −7.375 eV for Li–PD–NO_3_) or TFSI^−^ (−7.223 eV for Li–PEO–TFSI, −7.380 eV for Li–PD–TFSI). This indicates that the oxidative decomposition of Li–PEO–NO_3_ and Li–PEO–TFSI on the NCM811 surface is thermodynamically more favorable than that of Li–PEO, leading to the formation of an N,F‐rich CEI. In addition, the PD^+^ chain, featuring the positively charged quaternary ammonium group, exhibits superior oxidation resistance compared to the PEO segment. Consequently, it does not contribute additional organic decomposition products to the CEI, which helps maintain a stable and inorganic‐rich interphase. The specific composition of the derived CEI on the cycled NCM811 cathodes were analyzed by the time‐of‐flight secondary ion mass spectrometry (TOF‐SIMS) and XPS. In cells employing the PEO–PD–Li electrolyte, TOF‐SIMS depth profiles, along with 3D tomography and 2D mappings, demonstrate a homogeneous distribution of LiNO^−^ and LiF_2_^−^ species (Figure 5g,h), suggesting a CEI uniformly composed of LiN_x_O_y_ and LiF. XPS also provides direct evidence for the substantial presence of LiN_x_O_y_ and LiF species within the CEI (Figure 5i,j). The robust inorganic‐rich CEI not only exhibits high ionic conductivity but also suppresses the further oxidation of the electrolyte, thereby enhancing the interfacial stability of the cathode. Meanwhile, organic components (C_2_HO^−^ and C_2_H_3_O^−^) in the outer CEI could improve the elasticity and interfacial compatibility of the interphase [51].

Additionally, high‐resolution transmission electron microscopy (HRTEM) images demonstrate a non‐uniform and thick organic CEI film on the surface of NCM811 particle with PEO–Li electrolyte (Figure 5k), resulting from the decomposition of the organic solvation layer. On the contrary, a 3 nm thin inorganic‐rich CEI film is homogeneously formed by employing PEO–PD–Li electrolyte (Figures 5l, S31), where the interplanar distance of 0.231 and 0.267 nm can be assigned to the (111) plane of LiF and (111) plane of Li_2_O, respectively. The thin and robust inorganic CEI could improve the Li^+^ transport kinetics on the cathode side, and enhance the cycling stability of the high‐voltage cathode. Therefore, the PEO–PD–Li electrolyte can effectively improve the interfacial properties at both the anode and cathode, ensuring fast Li^+^ transport, compatibility with high‐voltage cathode and long‐term cycle stability of the battery.

### 2.4 Electrochemical Performance

**FIGURE 6:**
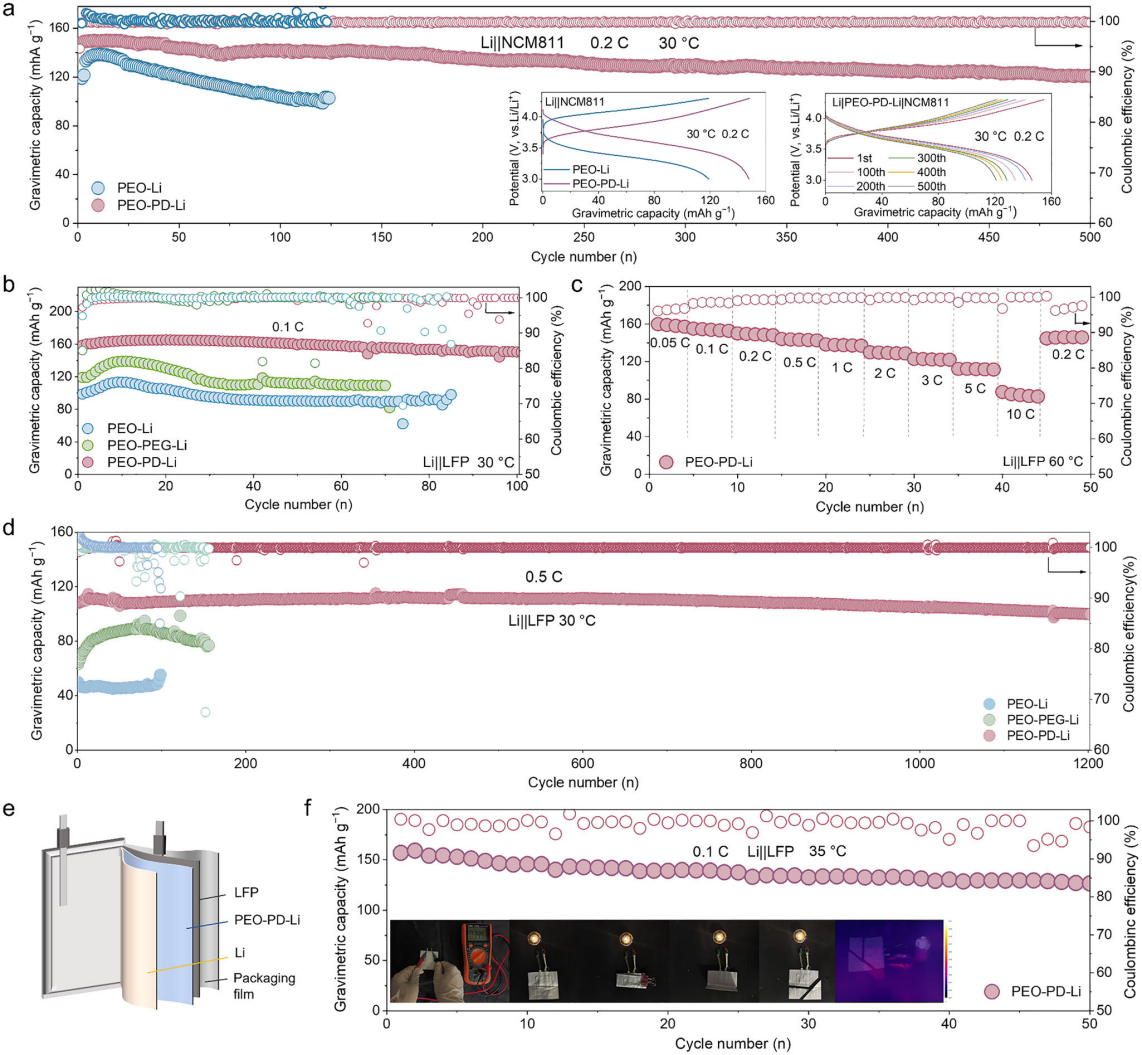
Electrochemical performance of the corresponding solid‐state lithium batteries. (a) Cycling performance of Li||NCM811cells with PEO–Li and PEO–PD–Li electrolytes at 0.2 and 30°C. Insets: (left) initial charge/discharge curves of both electrolytes at 0.2 C; (right) selected charge/discharge curves of PEO–PD–Li cell at different cycles. (b) Cycle performance at 0.1 C (30°C), (c) rate performance (60°C), (d) cycle performance at 0.5 C (30°C) for Li||LFP batteries assembled with the prepared polymer electrolytes. (e) The structure of the pouch cell. (f) The electrochemical performance of Li|PEO–PD–Li|LFP pouch cell. The inset corresponds to the safety tests during battery operation.

The performance of the PEO–PD–Li electrolyte was further assessed in lithium metal solid‐state batteries paired with NCM811 and LFP cathodes. No liquid was added during the battery assembly processes. The PEO–PD–Li electrolyte could endow the Li||NCM811 cell with ultra stable cycle performance near room temperature, demonstrating its potential in high‐voltage battery systems. Specifically, as evidenced by cyclic voltammetry (CV) and galvanostatic charge/discharge profiles, the Li|PEO–PD–Li|NCM811 cell exhibits significantly reduced electrochemical polarization and increased capacity due to the decreased energy barrier for fast Li^+^ ions transport (Figures 6a, S32). In addition, the Li|PEO–PD–Li|NCM811 cell demonstrates superior electrochemical performance under room temperature (30°C), exhibiting a discharge capacity of 146.3 mAh g^−1^ at 0.2C and an ultralow capacity decay rate of 0.035% per cycle over 500 cycles (Figure 6a). This highly stable cycling performance stems from the enhanced interface stability induced by the anion effect. In addition, when the operating temperature is raised to 60°C, the NCM811 paired with the PEO–PD–Li electrolyte exhibits a high capacity of 185.39 mAh g^−1^ at 0.1C, much higher than that with the PEO–Li electrolyte (158.7 mAh g^−1^, Figure S33), further ascertaining enhanced Li^+^ transfer kinetics of the PD‐modified electrolyte.

In addition, the significantly enhanced electrochemical performance is also observed in Li||LFP cells. Cyclic voltammetry (CV) and galvanostatic charge/discharge profiles reveal that the Li|PEO–PD–Li|LFP cell exhibits markedly reduced electrochemical polarization, as reflected by its exceptionally low voltage hysteresis (160 mV for PEO–PD–Li vs. 258 mV for PEO–PEG–Li and 286 mV for PEO–Li, Figures S34, S35). The related Li| PEO–PD–Li|LFP cell delivers a high initial capacity of 158.5 mAh g^−1^ at 30°C, and retains at 150.4 mAh g^−1^ after 100 stable cycles at 0.1 C with a high average Coulombic efficiency of 99.4% (Figure 6b). The EIS plots further reveal that the interfacial resistance of the Li|PEO–PD–Li|LFP cell undergoes only marginal increments after 50 cycles, confirming the exceptional stability of the electrolyte and interfaces (Figure S36). The solid‐state LFP cell also demonstrates superior rate capability under near‐ambient conditions (30°C, Figure S37). When the temperature rises to 60°C, the Li|PEO–PD–Li|LFP cell achieves enhanced rate performance, delivering specific capacities of 138.3, 129.9,122.9, 112.2, 87.3 mAh g^−1^ at 1, 2, 3, 5, 10 C rate, respectively, accompanied by the modest voltage difference and the long platform from 0.05 to 10 C (Figures 6c and S38). Notably, the long‐term cycle stability of the LFP solid‐state cell could truly be guaranteed at 0.5 C and 30°C (Figure 6d), with the capacity of 100.0 mAh g^−1^ after 1200 cycles, a low capacity decay rate of 0.0064% per cycle (retention rate of 92.3%), high average Coulombic efficiency of 99.94%, and stable charge/discharge plateaus (Figure S39). It starkly contrasts with the rapid capacity decay, limited cycle life, and high degradation rates observed in PEO–Li and PEO–PEG–Li counterparts (Figure 6d).

Moreover, to better examine the potential of PEO–PD–Li electrolyte, pouch cell with LFP cathode (4 cm^2^, 2.3 mg cm^−2^) and Li anode (6 cm^2^, 200 µm thick, Figure 6e) is trialed. The initial capacity could reach 156.3 mAh g^−1^ at 0.1 C and 35°C, and maintain at 126.6 mAh g^−1^ after 50 cycles with a capacity retention rate of 80.9% and a relatively small polarization voltage (Figures 6f and S40). More importantly, the assembled pouch cell could still work normally to light the bulb after various harsh treatments, such as folding for 90° and 180°, and cutting into pieces with no smoke, burning, or thermorunaway phenomenon occurred as observed from the infrared thermal image, verifying the safety and reliability of the as‐prepared solid batteries (the inserted images in Figure 6f). It is demonstrated that modifying PEO electrolytes with PD enables long‐term room‐temperature cycling stability of LFP and NCM811 batteries without adding liquid plasticizers, confirming the effectiveness of the as‐proposed multiscale strategy.

## 3 Conclusion

Through multiscale electrolyte regulation and anion coordination effects, this study successfully develops a high‐performance PEO‐based electrolyte for room‐temperature operation without the use of liquid plasticizers. By synergistically modulating both the polymer matrix and solvation structure, the proposed strategy effectively reduces the energy barriers for Li^+^ transport, desolvation, and interphase crossing, thereby significantly enhancing the overall ion transport kinetics both in the bulk electrolyte and at the interfaces. Additionally, the anion effect plays a crucial role in protecting both the anode and cathode by facilitating the formation of a Li_3_N/LiF‐rich SEI and a LiN_x_O_y_/LiF‐rich CEI, which enhances the interfacial stability and high‐voltage compatibility. As a result, both high‐voltage NCM811 and LFP cells demonstrate exceptional cycling stability at room temperature. This work provides invaluable insights into SPE design and interfacial engineering, offering a promising pathway for developing high‐performance polymer‐based solid‐state batteries.

## Conflicts of Interest

The authors declare no conflicts of interest.

## Supporting information




**Supporting File**: anie71547‐sup‐0001‐SuppMat.docx.

## Data Availability

The data that support the findings of this study are available on request from the corresponding author. The data are not publicly available due to privacy or ethical restrictions.
