# Supplementary material for: Multiscale Engineering of PEO Electrolytes for High‐Voltage and Ultrastable Solid‐State Lithium Batteries With Exceptional Room‐Temperature Performance
Source: Angew Chem Int Ed Engl. 2026 Feb 18;65(14):e23382. doi: 10.1002/anie.202523382 (PMC13023716; doi:10.1002/anie.202523382)
Supplement: Supplementary file 1 — Supporting File: anie71547‐sup‐0001‐SuppMat.docx. [file ANIE-65-e23382-s001.docx]

Supporting Information

**Multiscale Engineering of PEO Electrolytes for High-Voltage and Ultrastable Solid-State Lithium Batteries with Exceptional Room-Temperature Performance**

Xuefan Liu,^‡^ ^[a]^ Bowen Zhang,^‡^ ^[a]^, Congcong Zhang,^[a]^ Xueyu Zhou,^[a]^ Xu Liu,^[a]^ Teng Liu,^[a]^ Shifeng Hou,^[a]^ Qifeng Zheng,^[c]^ Lu Wang,*^[a]^ Linglong Kong,*^[b]^ Shanqing Zhang,*^[c]^

^a^Key Laboratory of Low-Carbon and Green Agriculture Chemistry in Universities of Shandong, College of Chemistry and Material Science, Shandong Agricultural University, Tai’an, Shandong 271018, China

^b^State Forestry and Grassland Administration Key Laboratory of Silviculture in Downstream Areas of the Yellow River, College of Forestry, Shandong Agricultural University, Tai’an, Shandong 271018, China

^c^Institute for Sustainable Transformation, School of Chemical Engineering and Light Industry, Guangdong University of Technology, Guangzhou, Guangdong 510006, China.

*Corresponding authors. E-mail: [luwang@sdau.edu.cn](mailto:luwang@sdau.edu.cn) (L. Wang), [linglongkong@sdau.edu.cn](mailto:linglongkong@sdau.edu.cn%20) (L. Kong), s.zhang@gdut.edu.cn (S. Zhang).

^‡^These authors contributed equally to this work.

Table of Contents

1. Experimental Section………………………………………………………………………..……..3-5
2. Supporting Figures…………………………………………………………………………………6-24
3. Supporting Tables……………………………………………………………………………….....25
4. References…………………………………………………………………………………………..26

Experimental Section

1. **PD and the composite electrolyte synthesis.**

The 2-(Dimethylamino)ethyl methacrylate (DMAEMA, 99%, Aladdin) modified polyethylene glycol (PEG, Mn≈6000 g mol^−1^, Aladdin) was polymerized according to the literature^[1]^, by dissolving 5.0 g DMAEMA, 5.0 g PEG and 1 mL of nitric acid (70wt%, Sinopharm) in 70 mL of deionized water, and deoxygenating with nitrogen stream for 1 h at 55 °C and continual stirring, followed by dropwise adding 3 mL of HNO_3_ aqueous solution (0.5 mol L^−1^) containing 6 mmol ammonium cerium nitrate (99.99%, Aladdin) and keeping stir under nitrogen atmosphere for 4 h. The obtained products were further dialyzed by using deionized water in a dialysis bag (Spectrum Laboratories, Inc.) with a molecular weight cutoff of 3500 for 7 days to obtain the PD additive.

The corresponding solid polymer electrolytes (SPEs) were prepared through the common solution casting technics.^[2]^ Specifically, 0.62 g PEO (Mv≈600000 g mol^−1^, Aladdin), 0.2 g PD, and 0.38 g of lithium bis(trifluoromethanesulfonyl)imide (LiTFSI, 98%, J&K) powders were dissolved in anhydrous acetonitrile (10 mL, 99.9%, YongDa Chemistry) after vigorous stirring at 60 °C with the [EO]:[Li] molar ratio of 11:1. The homogeneous solution was cast onto a homemade ultrathin porous bacterial cellulose membrane (Φ40 mm × 7 μm), then dried at room temperature for 12 h and finally vacuum-dried at 60 °C for 12 h, designated as PEO-PD-Li. The cellulose membrane substrate was fabricated by first filtering a bacterial nanocellulose dispersion (0.05%, 5 mL) under reduced pressure, followed by a stepwise drying process (12 h at 60 °C and then 3 h at 103 °C).

Similarly, the control solid electrolyte films were made via the same procedure by adjusting the amount of PD or substituting with the PEG (Mn≈8000 g mol^−1^, Aladdin) (marked as PEO-PEG-Li) in the fixed [EO]/[Li] ratio. The PEO-PEG-Li+LiNO_3_ electrolyte consists of 0.62 g of PEO, 0.2 g of PEG, 0.014 g of LiNO_3_, and 0.38 g of LiTFSI. The obtained electrolyte membranes were cut into discs (Φ=19 mm) for coin cells, then reserved in an Ar-filled glove box with extremely low oxygen and moisture concentrations both below 0.01 ppm (Super, Mikrouna).

1. **Structure characterization**

The structure and morphology of the obtained solid-state electrolytes and the related electrodes were confirmed by using the X-ray diffraction (XRD, Rigaku Smartlab) in the 2θ range of 10~60°, field emission scanning electron microscopy (FESEM, Hitachi SU8010) equipped with energy dispersive spectrometer, high-resolution transmission electron microscopy (HRTEM, JEM-2100F) and atomic force microscopy (AFM, FM-Nanoview), respectively. Their chemical states were further examined by employing X-ray photoelectron spectroscopy (XPS, Thermo Escalab 250Xi), Fourier transform infrared spectroscopy (FTIR, Thermo Nicolet, 400 ~4000 cm^−1^), Raman spectroscopy (DXR2xi) with a 532 nm laser, and ^1^H nuclear magnetic resonance spectroscopy (NMR, Bruker AVANCE III), respectively. The differential scanning calorimetry (DSC, Netzsch DSC214) was conducted from −80 °C to 80 °C in determining the T_g_ of the electrolyte. The crystallinity of different electrolytes was evaluated through the polarizing microscopy (Zeiss Axio Scope A1). The interfacial property of the cycled cathode was confirmed by time-of-flight secondary ion mass spectrometry (TOF-SIMS, TOF.SIMS 5-100, IONTOF), which was operated in negative ion mode using a Bi cluster primary-ion gun (30 keV, i=45°) for analysis and a Cs^+^ sputter gun (1 kV, 80 nA, i=45°) for depth profiling on the regions of 100 x 100 μm^2^ and 300 x 300 μm^2^, respectively, with a 128 x 128 pixels on the mappings.

1. **Electrochemical performance tests**

Electrochemical impedance spectroscopy (EIS) of the assembled stainless steel |electrolyte membrane| stainless steel simulated cells (2025-type) was conducted on the electrochemical workstation (CHI660e, CH Instruments, Inc.) from 30 °C to 70 °C with the 10 °C interval and the set frequency range (0.01 Hz~100 kHz) in the amplitude of 5 mV to obtain the impedance values and apply in the formula ($\sigma=\frac{L}{RS}$) for the ionic conductivity calculation of the electrolyte membrane, where R, S and L referred to the measured impedance, area and thickness of the electrolyte membranes, respectively. The activation energy for the polymer electrolytes was analyzed using the Vogel–Tamman–Fulcher (VTF) equation, which is suitable for describing ion transport coupled with segmental motion above the glass transition temperature (Tg)^[3-5]^. The VTF equation is expressed as:$\sigma=\frac{A}{T^{0.5}}*exp(\frac{E_{a}}{R\left( T\text{-}T_{0} \right)}\text{)}.$

where σ is the ionic conductivity, *A* is the pre-exponential factor, *E*ₐ is the VTF activation energy for ion migration, *R* is the gas constant, and *T* is the absolute temperature. The parameter *T*_0_ is the Vogel temperature, which is typically 50 K below the experimental *T*_g_ for polymer electrolytes and reflects the theoretical temperature at which segmental motion would cease^[3,4]^. The nonlinear curve fittings were performed to obtain the VTF parameters.

The electrochemical stability window of the solid electrolyte was evaluated by using liner sweep voltammetry (LSV) in the 2025-type coin cells with SS as working electrode and Li foil as counter and reference electrode. This test was carried out on the CHI660e-type electrochemical workstation at a scan rate of 0.1 mV s^−1^ from 0 V to 6.0 V (vs. Li/Li^+^). The Li^+^ transference number of the electrolyte film at 30 °C was acquired through the direct current (DC) polarization method in the Li||Li symmetric cells (CR2025 type, Φ_Li_= 14 mm), and set the polarization voltage (ΔV) as 10 mV.

The initial current (I_0_), steady state current (I_s_), the pristine/post interfacial impedance (R_L0_/R_Ls_) and charge transfer impedance (R_b0_/R_bs_) from the EIS spectra (0.01 Hz~ 100 kHz, 10 mV) were recorded and applied in the calculation formula

($t_{{Li}^{+}}=\frac{I_{s}R_{bs}（\Delta V-I_{o}R_{Lo}）}{I_{o}R_{bo}（\Delta V-I_{s}R_{Ls}）}$).

Electrochemical floating experiments were performed using Li||NCM811 cells with PEO-Li and PEO-PD-Li electrolytes. The cells were charged to 4.0 V at 0.05 C (1 C = 220 mA g^−1^) and then held at gradually higher voltages.

The exchange current density was measured at 30 °C by scanning from −0.25 V to 0.25 V at a scan rate of 1 mV s^−1^ and calculated based on the Tafel equation. The desolvation energy barrier (ΔE_desolvation_) and Li^+^ transport barriers through the electrode-electrolyte interface (ΔE_SEI_) can be calculated from the Arrhenius equation ($\sigma=AT^{-1}e^{-\frac{E_{a}}{RT}}$).

The Li plating/stripping experiments on Li||Li symmetric cells were performed at various current density on CT-4008 battery test system (Neware), including a long cycle at 0.1 mA cm^−2^/0.1 mAh cm^−2^. The LiFePO_4_ and NCM811 cathodes were fabricated with a mass ratio of 80% active material, 10% Super P, 5% PEO, and 5% LiTFSI. The slurry was stirred at 800 rpm for 12 h, coated onto an Al foil, dried at 60 °C for 12 h, and cut into discs (Φ = 10 mm) for the coin cells. These cathodes were directly used to assemble CR2025 coin cells and pouch cells without the addition of liquid electrolyte. The active materials loading was controlled at 1~2 mg cm^−2^ for coin cells and 2~3 mg cm^−2^ for pouch cells.

The corresponding cycle and rate performance of the assembled all-solid-state lithium metal batteries (ASSLMB) was gained on battery testing instruments (CT-4008, Neware) at different C (LFP, 1 C=170 mA g^−1^; NCM811, 1 C= 220 mA g^−1^) rates after shelving at 30 °C for 12 h. The total process of battery assembly was implemented in the Ar-filled glove box (H_2_O and O_2_< 0.01 ppm). All the electrochemical tests were repeated no fewer than three times.

**4. Computational Method**

Molecular dynamics simulations were performed with GROMACS 2024.2^[6]^. The OLPS-AA force field was used. The model molecules were optimized with Gaussian 16 at the B3LYP-D3/6-31G* level. The topologies for the PEO and the DMAEMA model molecules were created using the LigParGen web server^[7]^. The topologies of the other molecules were adopted from ref^[8]^. The 1.2* CM5 atomic charge was used (calculated with Multiwfn 3.8 dev^[9]^). The atomic charges for ions were scaled in a factor of 0.8. A cubic 50 nm * 50 nm * 50 nm periodic box was used to contain the model molecules. Energy minimizations were performed using the conjugate gradient algorithm (tolerance = 100 kJ·mol^−1^ nm^−1^). Pre-equilibrium phase simulations were performed using the NPT ensemble with the Berendsen thermostat at 598.15 K for 50 ns and then 303.15 K for 50 ns. The Berendsen barostat was set to be 1.01325 bar. The PME cutoff scheme was used for the coulomb and van der Waals interaction with 1.0 nm cutoff radius for both. The production phase simulations were performed with an NVT ensemble at 303.15 K using the Velocity-rescale thermostat for 10 ns. Simulation in the electric field (0.1 V/nm in the z-direction) was carried out for 10 ns. The step size was set to be 1 ps for the pre-equilibrium phase and 2 ps for the production phase.

As for density functional theory (DFT) calculations, geometric optimizations and energy calculations were performed with Gaussian 16 C01^[10]^. B3LYP^[11]^ functional was used. The 6-31+G(d) basis set was used in geometric optimizations and frequency calculations. Grimme’s dispersion correction^[12]^ with Becke-Johnson damping^[13]^ was applied. Grimme’s quasi-harmonic approximation^[14]^ was applied to correct the entropy contribution from low-frequency vibrational modes to the Gibbs free energies. 0.9829, 1.0053 and 1.0080 was used as the frequency scaling factor for zero-point energy, U(T)-U(0), and S(T)^[15]^. The thermodynamics were calculated at 303.15 K (30 °C). The IEFPCM implicit solvent model^[16]^ was included in the geometric optimizations. Diethyl ether was chosen as the solvent for the IEFPCM model based on the following rationale. The relative dielectric constant of PEO is reported to be in the range of 2.6 to 3.8^[17]^, which is comparable to that of diethyl ether (~4.3). In addition, diethyl ether shares similarity with PEO in terms of chemical composition, thus ensuring the rationality of the non-electrostatic component of the solvation correction.

The free volume fraction (*f*) is a dimensionless parameter that quantifies the proportion of unoccupied space within a molecular dynamics (MD) simulation box. It is computed according to the following method: For a specific grid (*x,y,z*)d*A* in the MD simulation box, if its distance from any atom is within the van der Waals radius of this atom, this grid is considered occupied. The sum of all unoccupied grid area is the free volume. The calculation of the free volume was performed using the Multiwfn code (version 3.8 dev), employing the formula: $f=\frac{\left( N_{total}-N_{occ} \right)\times a^{3}}{L_{x}\times L_{y}\times L_{z}}$.

Where, *f* is the free volume fraction, *N*_total_ is the total number of cubic grid points, *N*_occ_ is the number of occupied grid points, *a* is edge length of a single cubic grid point, *L_x_, L_y_, L_z_* are the lengths of the simulation box along the three axes. A grid size of 0.25 Angstrom was used. Periodic boundary condition was taken into account to gain accurate results for areas near the boundary. When visualizing the isosurface of free volume regions, a smoothed grid data was used, in which a Gaussian function with full width at half maximum (FWHM) of 1.8 was employed as the switching function that defines the smoothed boundary. Note that this smoothing is only implemented during the visualization to improve the visualization quality but does not influence the calculated free volume.

Supporting Figures


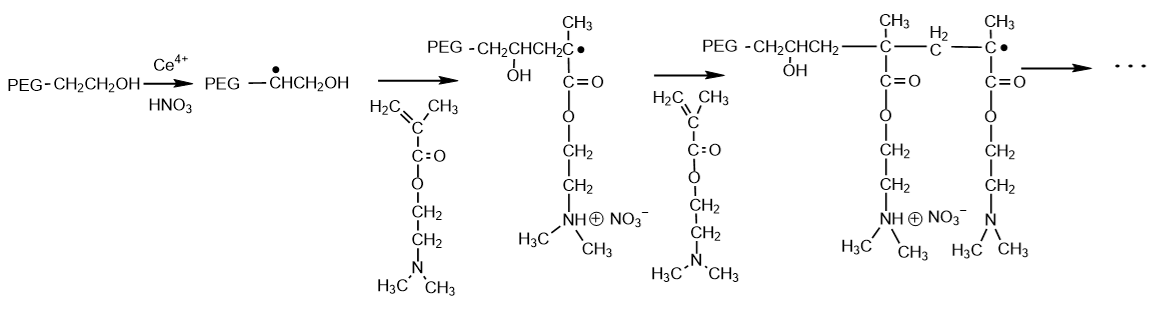


Figure S1. Initiation and propagation steps in the free radical polymerization of poly(ethylene glycol)-poly(2-dimethylaminoethyl methacrylate) nitrate.


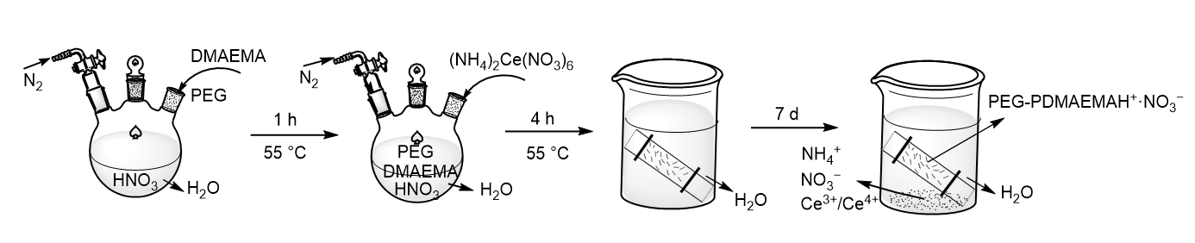


Figure S2. Schematic illustration of the synthesis reaction for the PD powders.


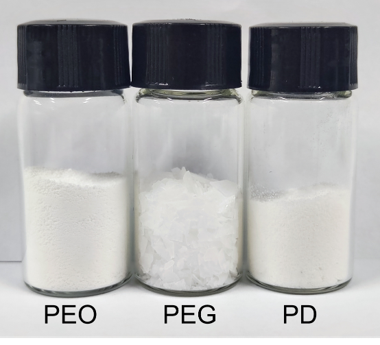


Figure S3. The photographs of PEO, PEG and PD powders.


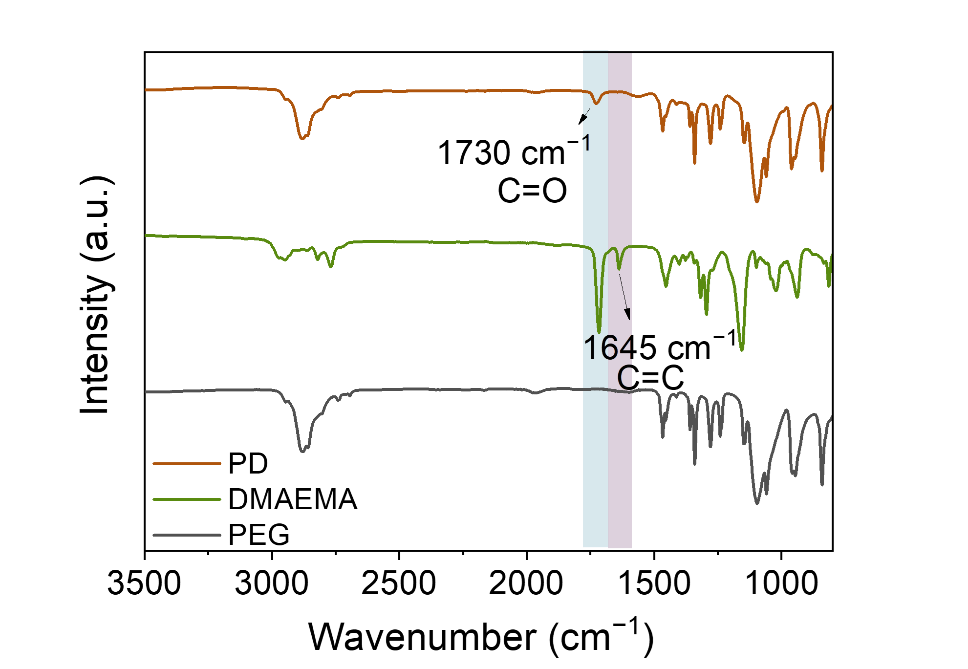


Figure S4. FTIR spectra for DMAEMA, PEG and PD powders.


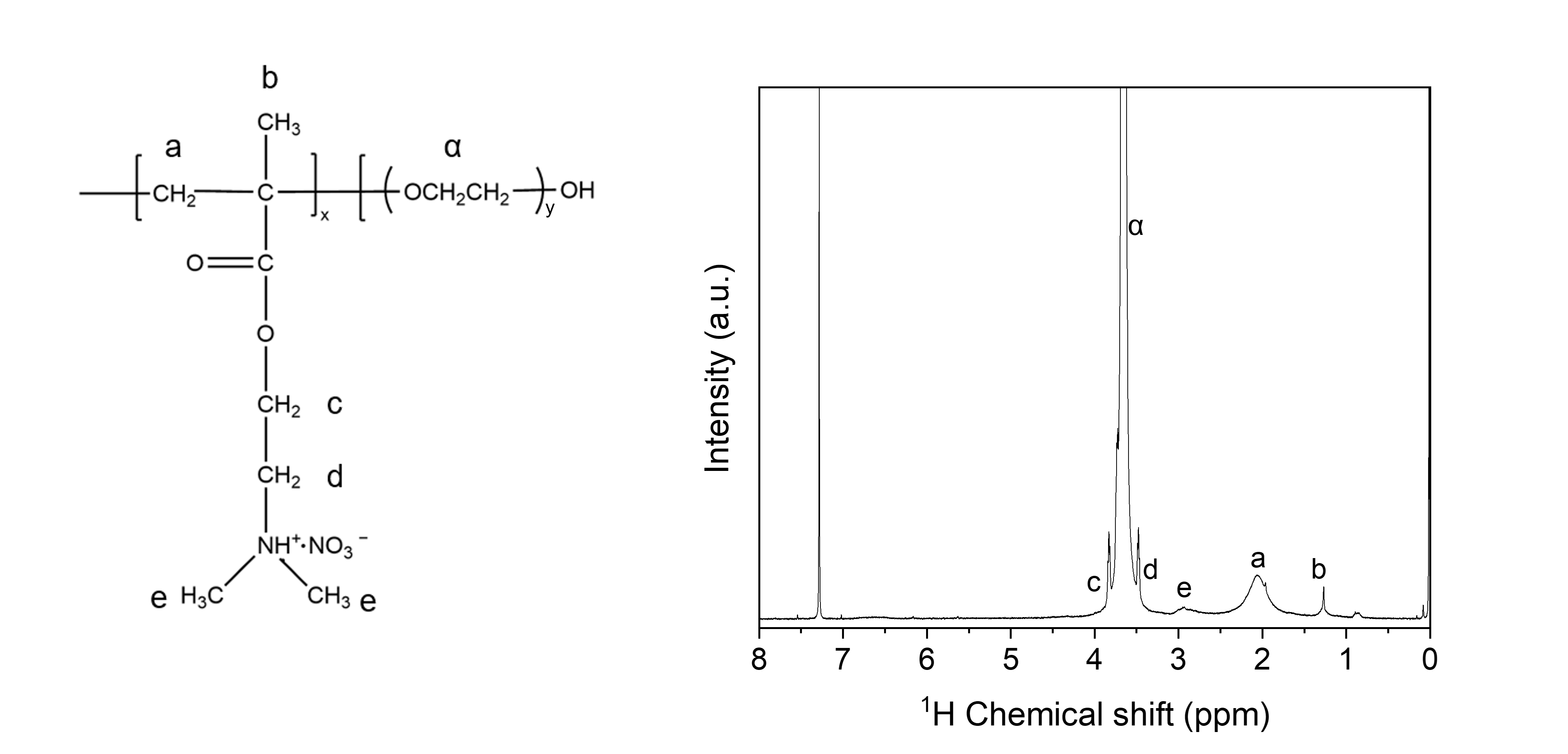


Figure S5. The molecular structure and ^1^H NMR spectrum of PD.


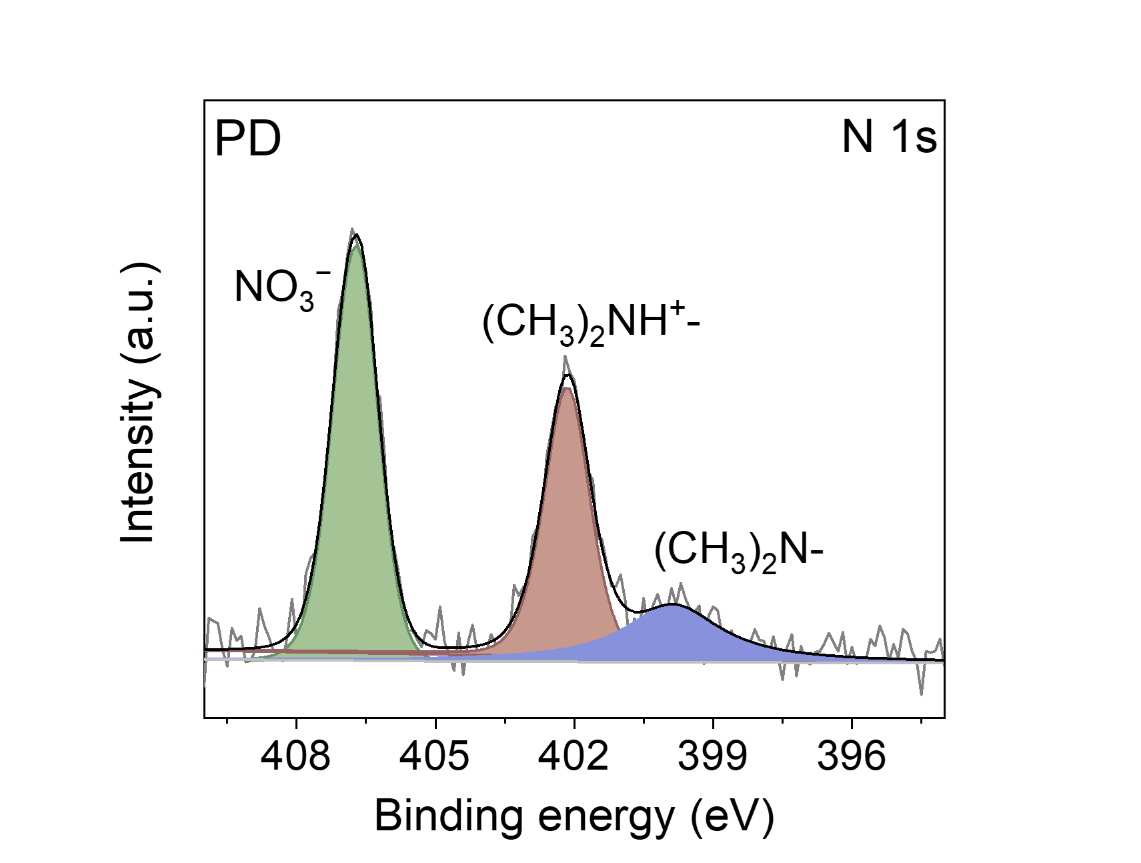


Figure S6. XPS spectrum of N 1s for PD powders.


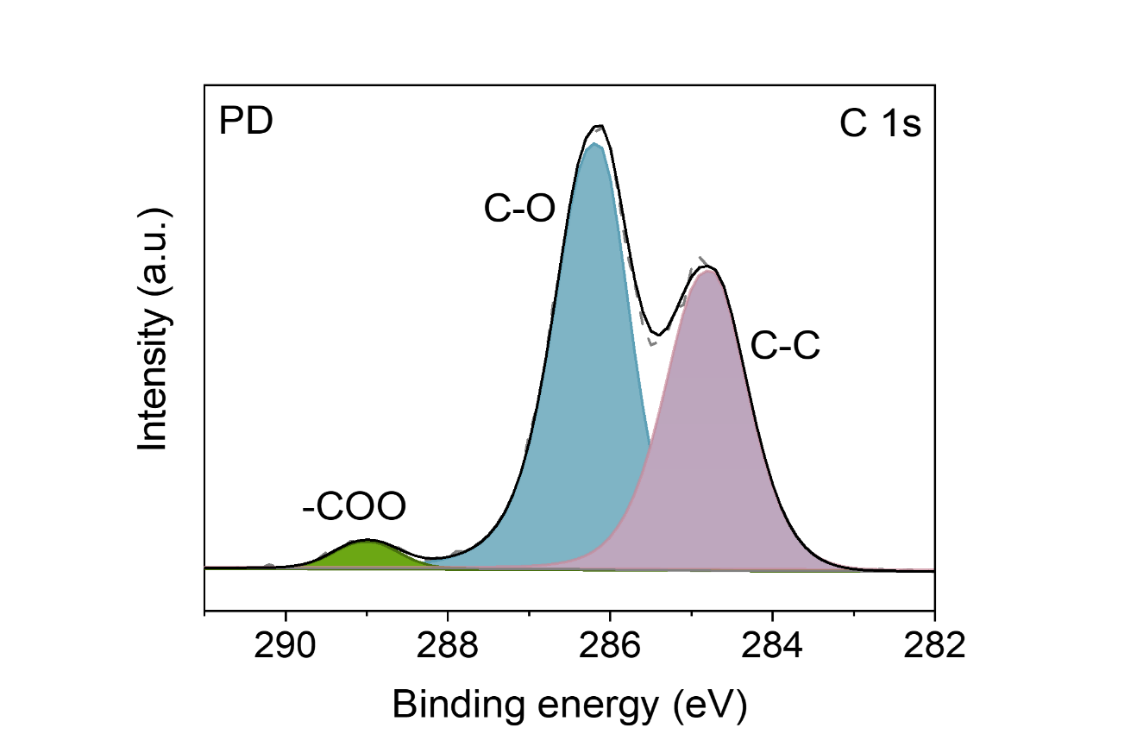


Figure S7. XPS spectrum of C 1s for PD powders.


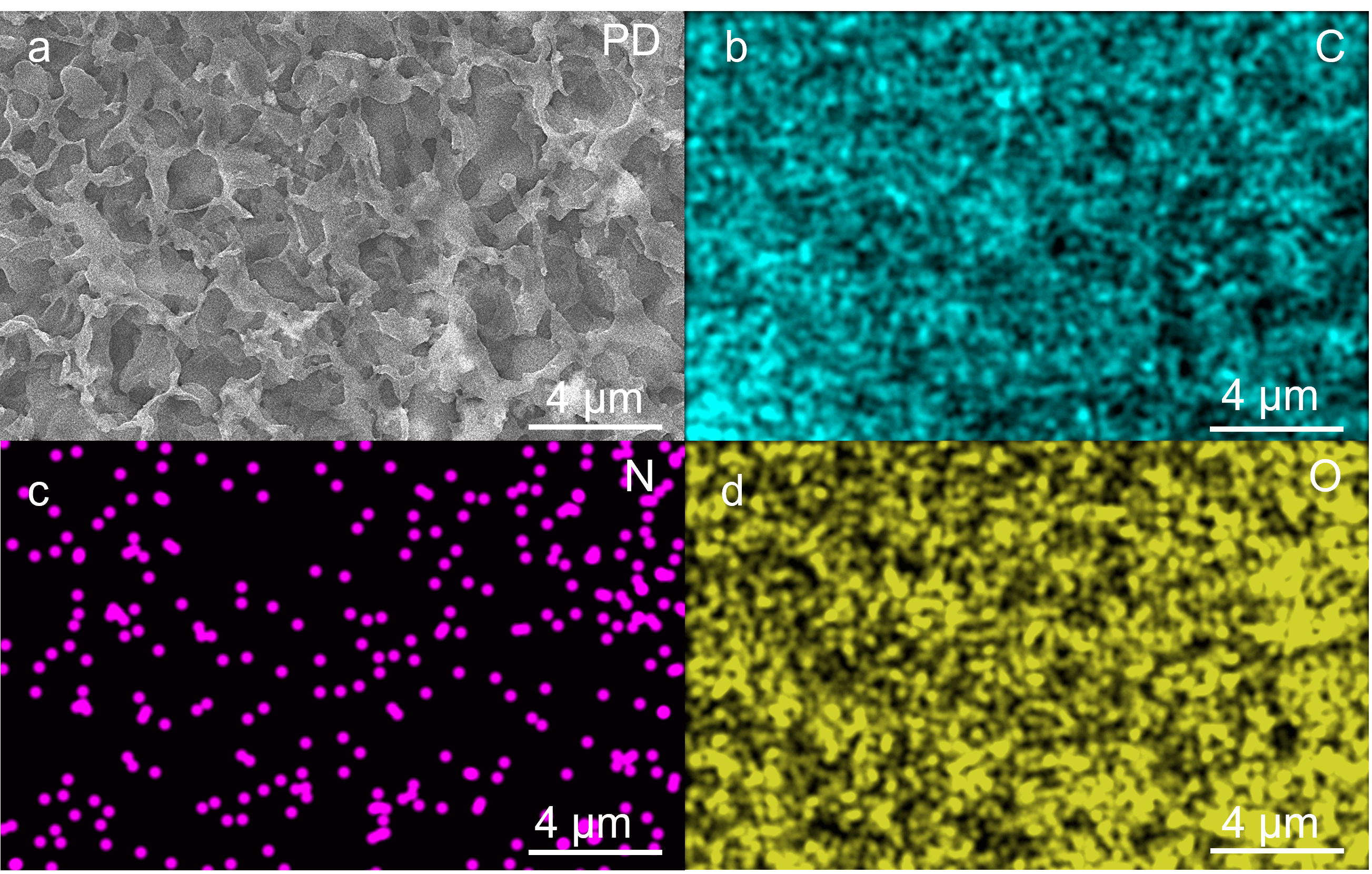


Figure S8. EDS mappings of the PD powders.


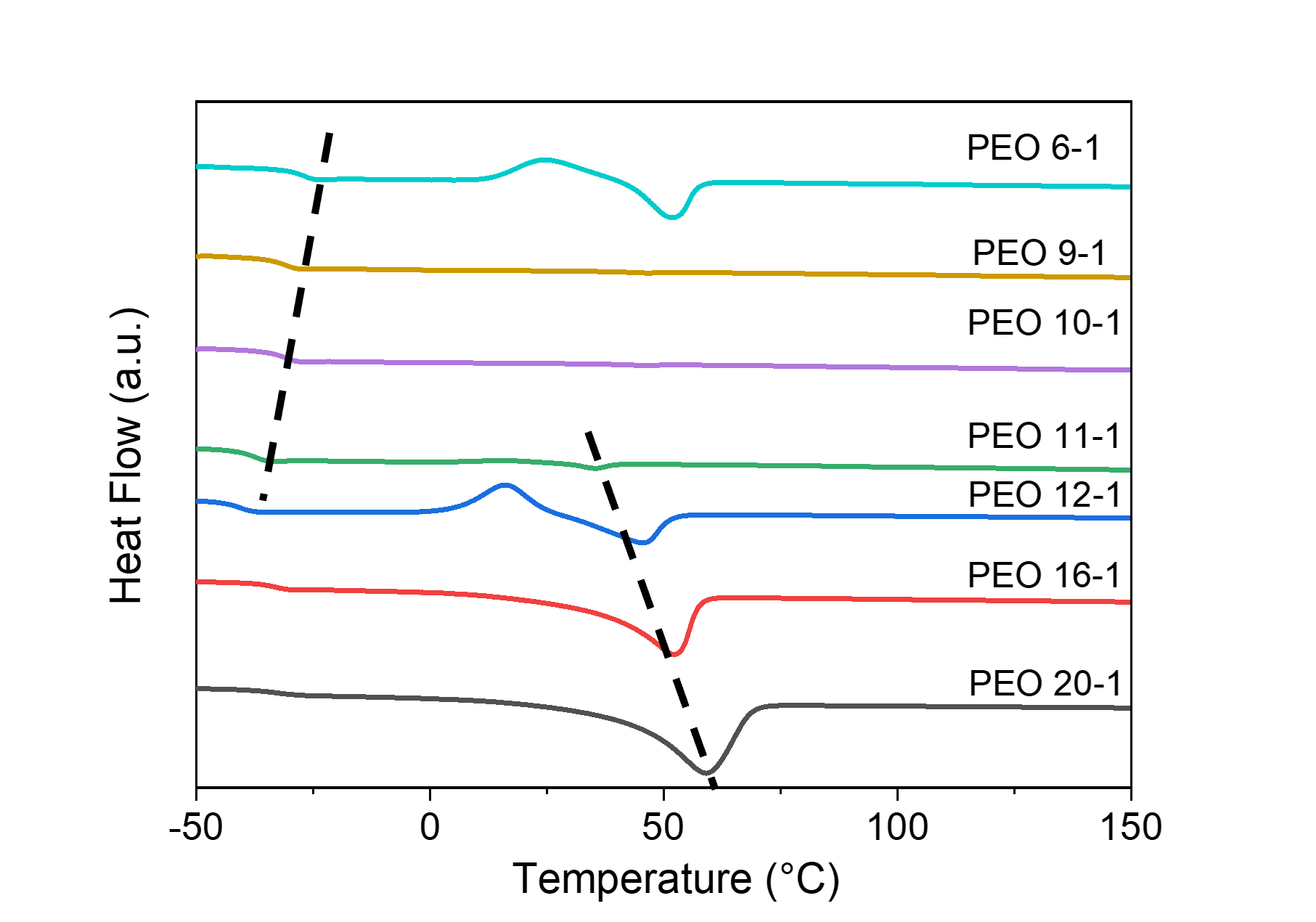


Figure S9. DSC curves of the PEO electrolyte with different LiTFSI ratios, ranging from 20:1 to 6:1 (EO:Li molar ratio).


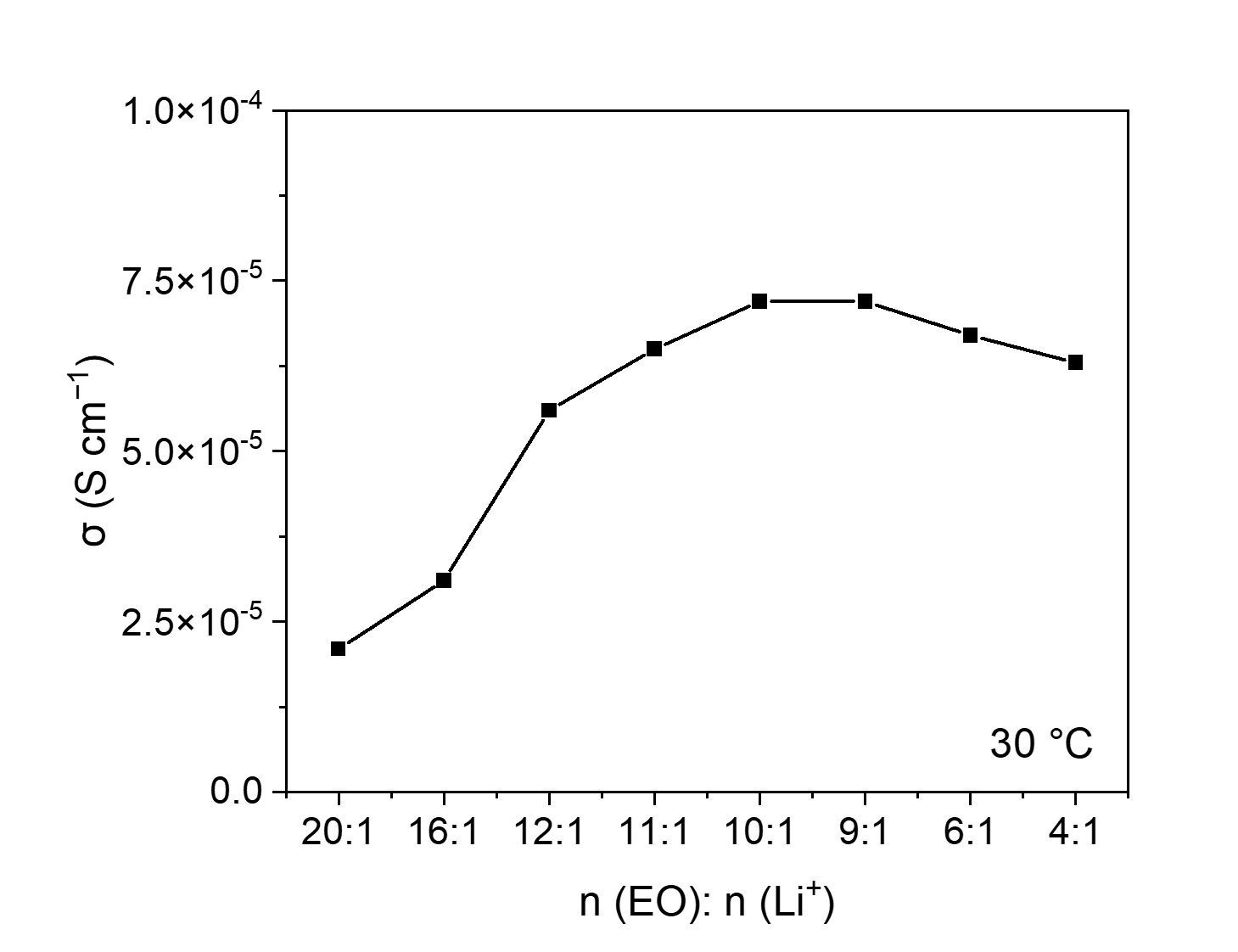


Figure S10. The Li^+^ ion conductivity of the PEO electrolyte with the increase of LiTFSI ratio. Given that the electrolyte with an EO:Li ratio of 11:1 exhibited the relatively lowest glass transition temperature (*T*_g_) and highest Li^+^ ion conductivity, this lithium salt concentration was chosen for the further investigation.


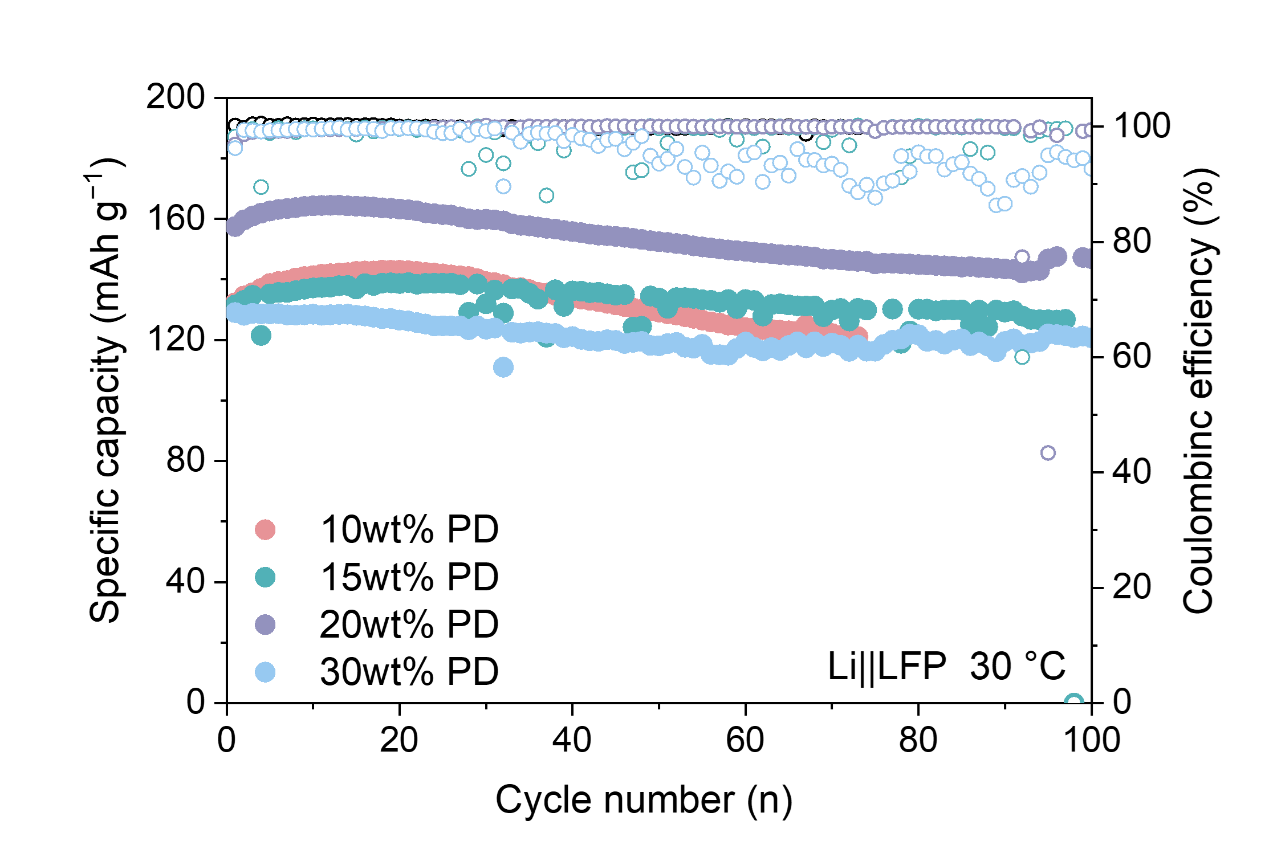


Figure S11. Cycle performance of the Li||LFP cells using PEO electrolytes with different PD addition amounts at 30 °C.


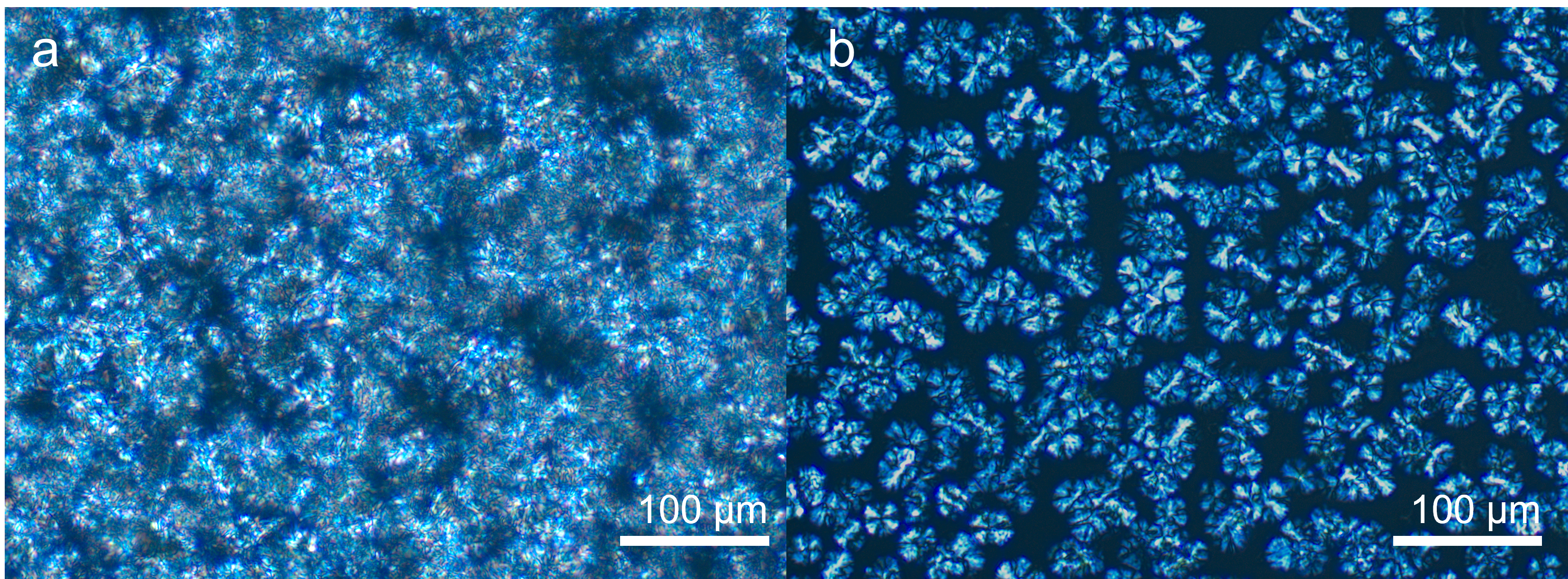


Figure S12. Polarized light microscope images of (a) PEO and (b) PEO electrolyte with an EO:Li molar ratio of 20:1.


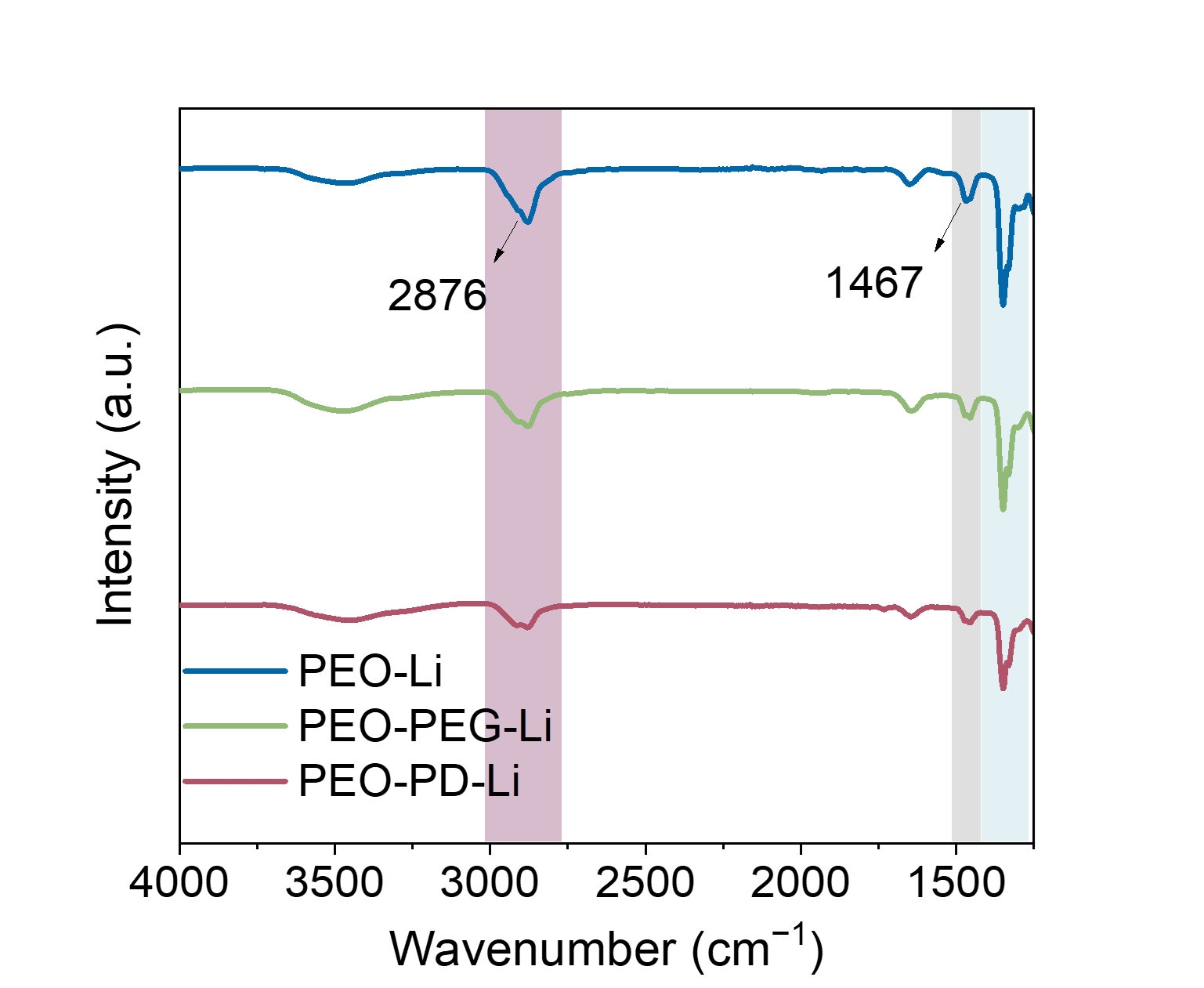


Figure S13. FTIR spectra for the PEO-Li, PEO-PEG-Li and PEO-PD-Li electrolytes.


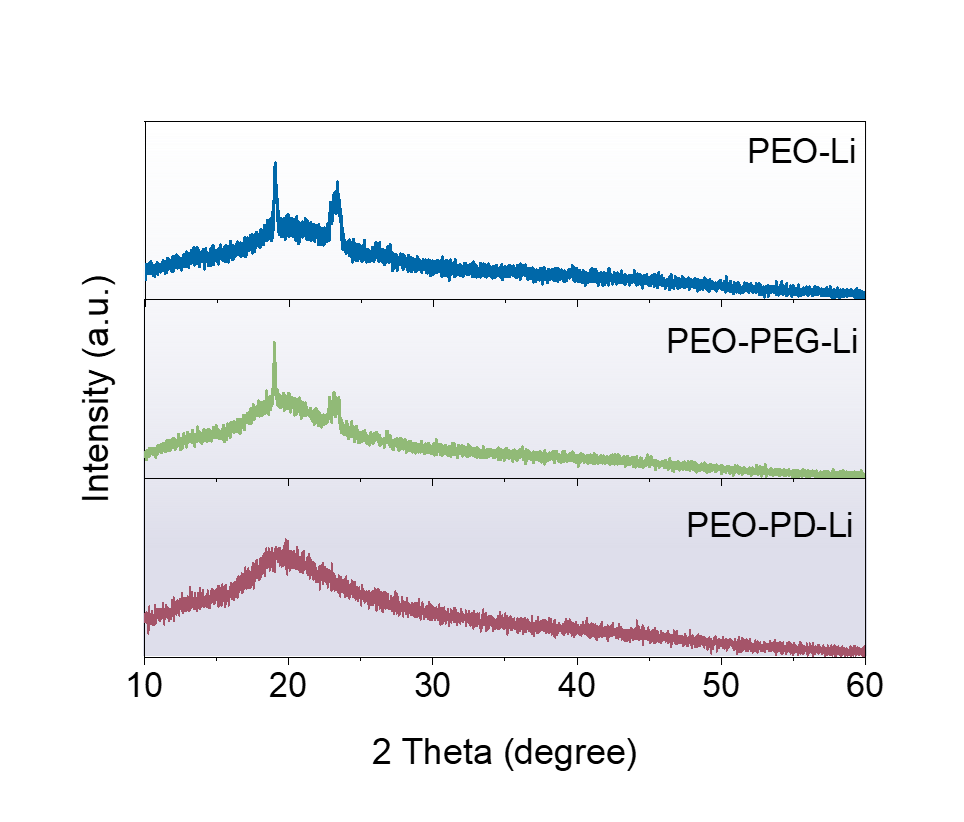


Figure S14. XRD patterns for the PEO-Li, PEO-PEG-Li and PEO-PD-Li electrolytes.


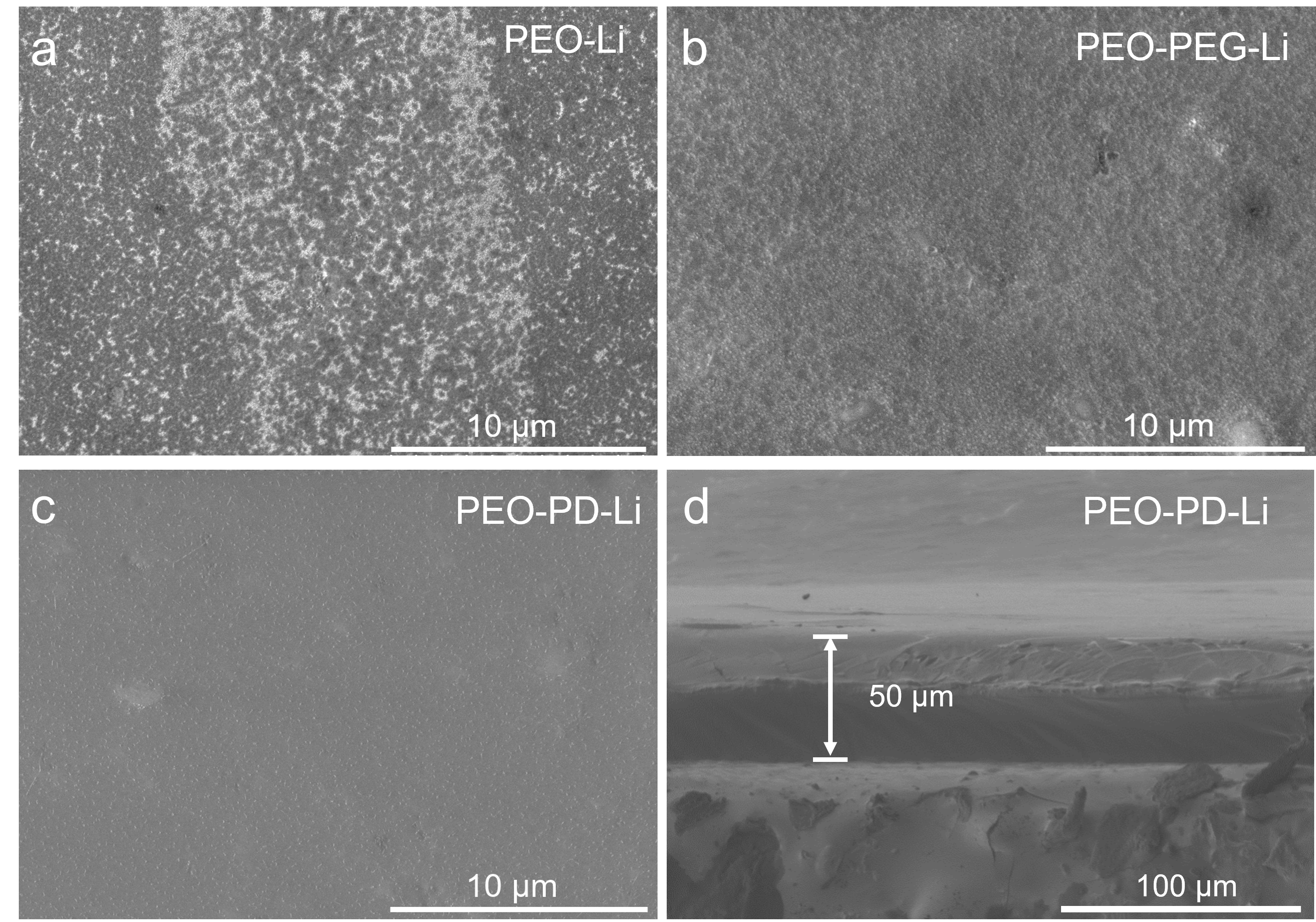


Figure S15. SEM images of the (a) PEO-Li, (b) PEO-PEG-Li, and (c,d) PEO-PD-Li electrolytes.


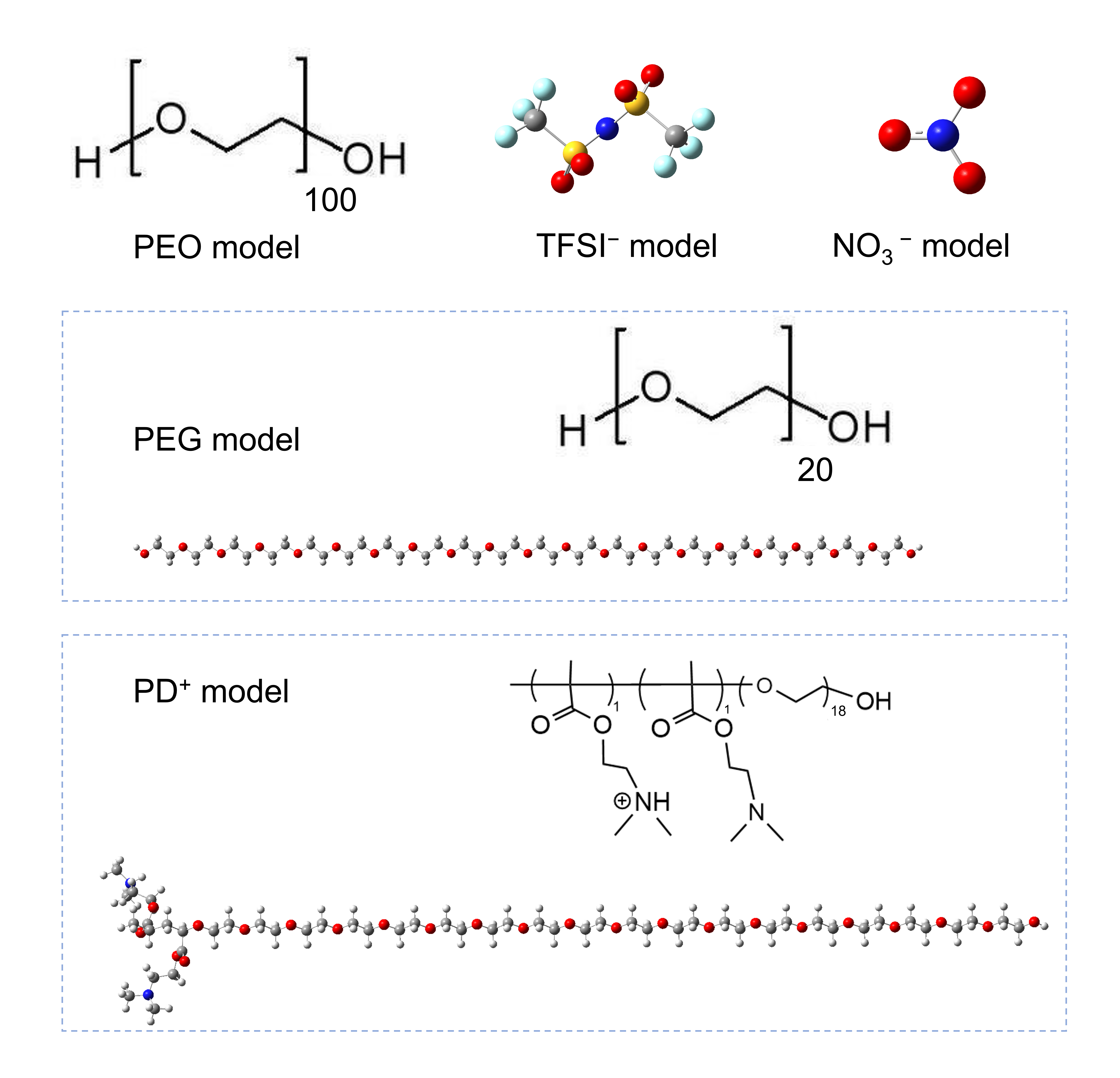


Figure S16. Molecule models used in MD simulations. Each PD^+^ chain has one positive quaternary amine group and one tertiary amine group.


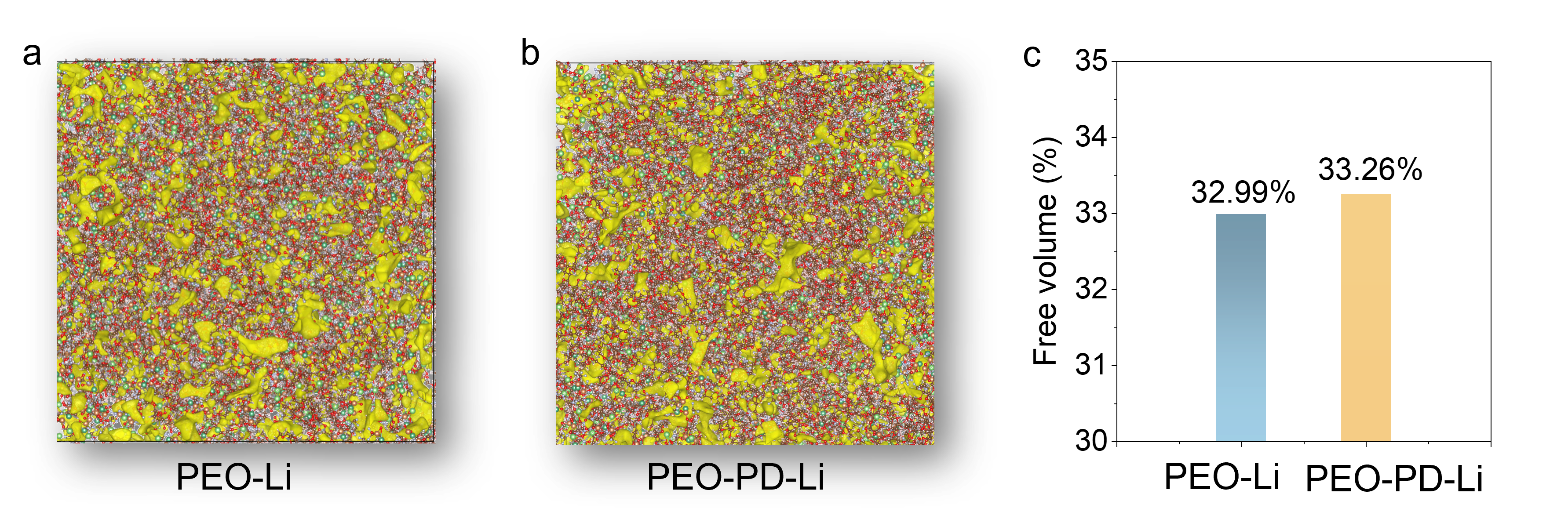


Figure S17. MD simulation snapshots of free volume (the yellow part) for (a) PEO-Li and (b) PEO-PD-Li electrolyte. (c) Free volume fraction comparison for PEO-Li and PEO-PD-Li electrolyte.


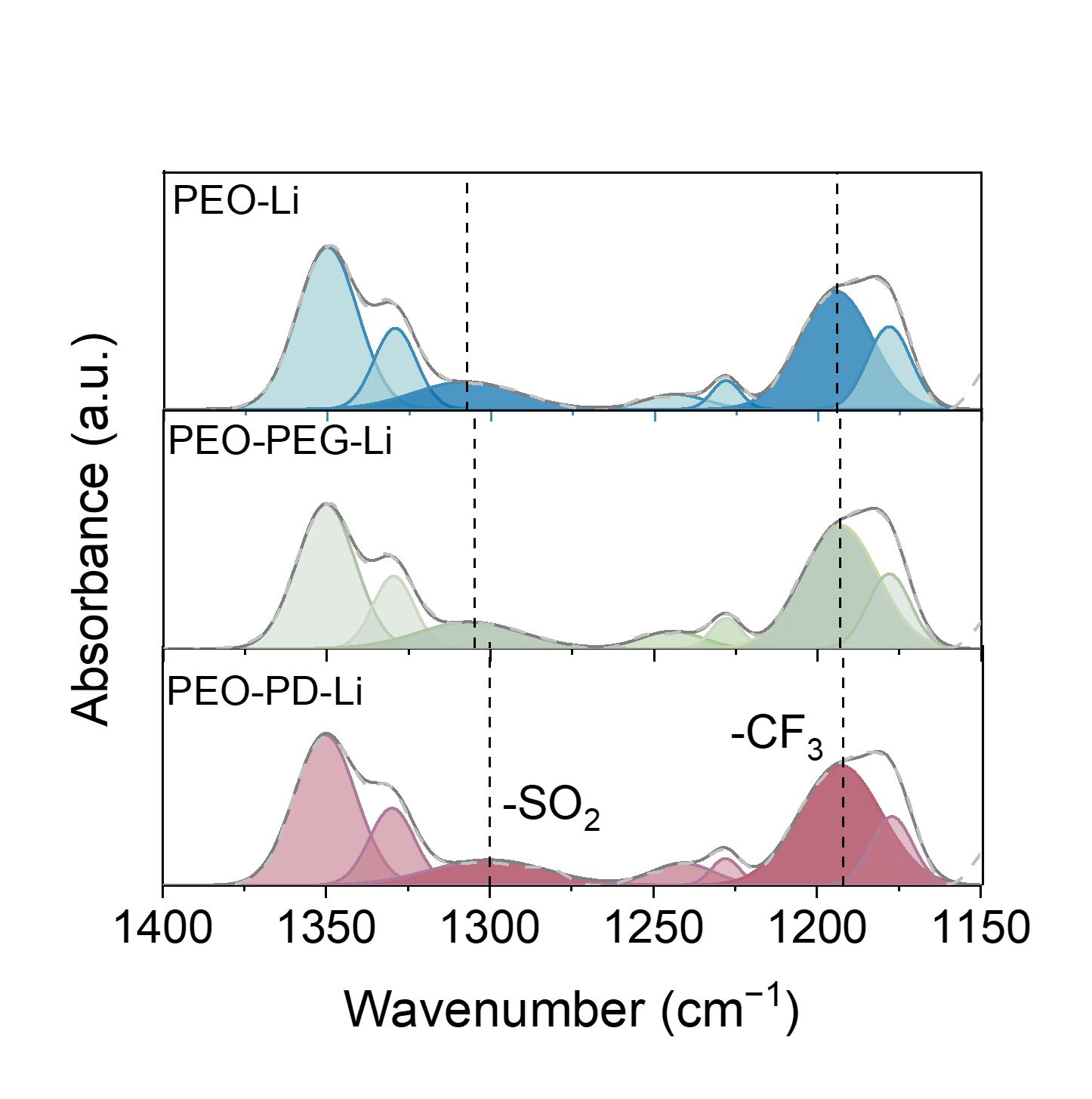


Figure S18. FTIR spectra for the PEO-Li, PEO-PEG-Li, PEO-PD-Li electrolytes.


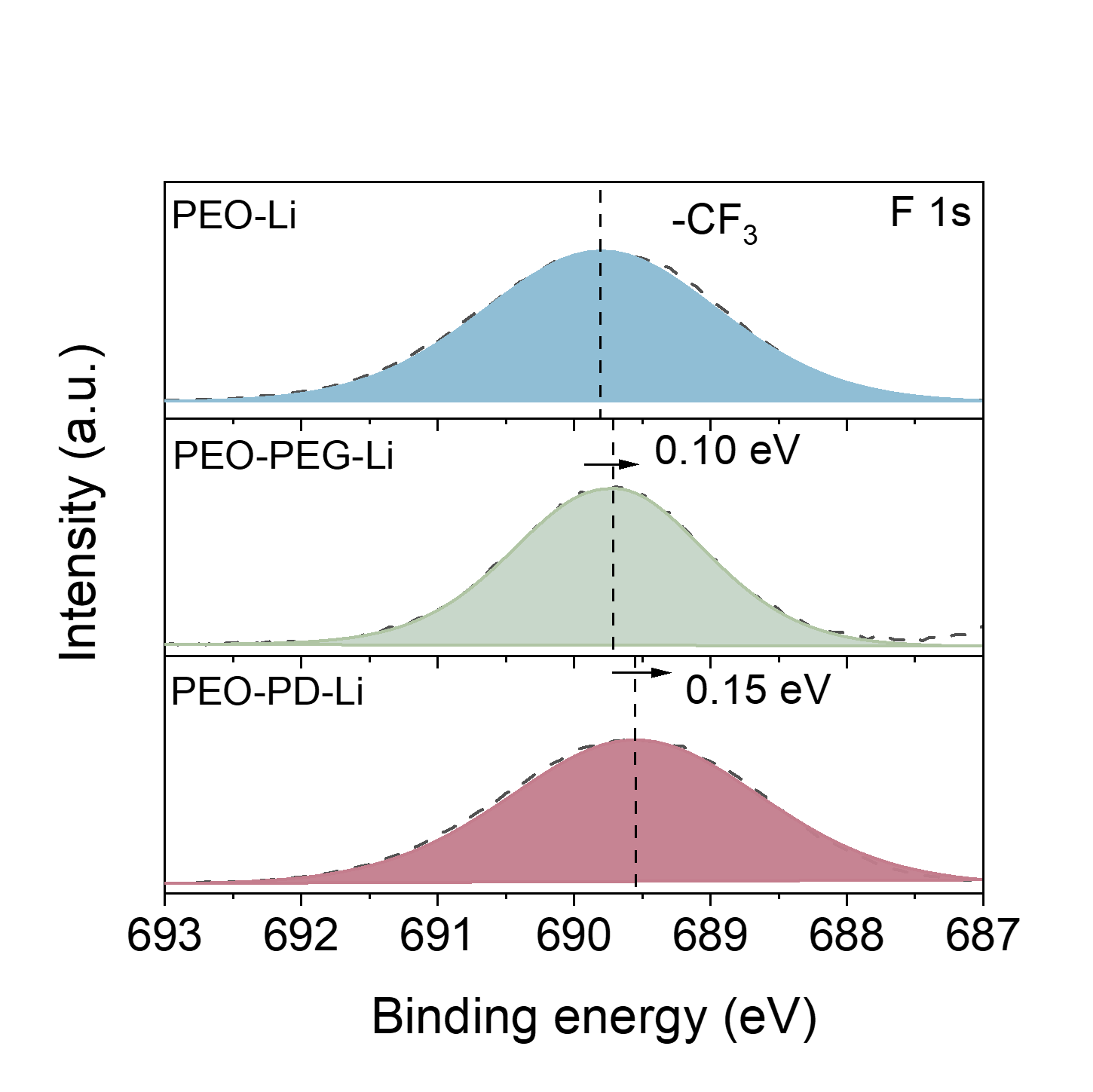


Figure S19. F 1s XPS spectra for the PEO-Li, PEO-PEG-Li, PEO-PD-Li electrolytes.


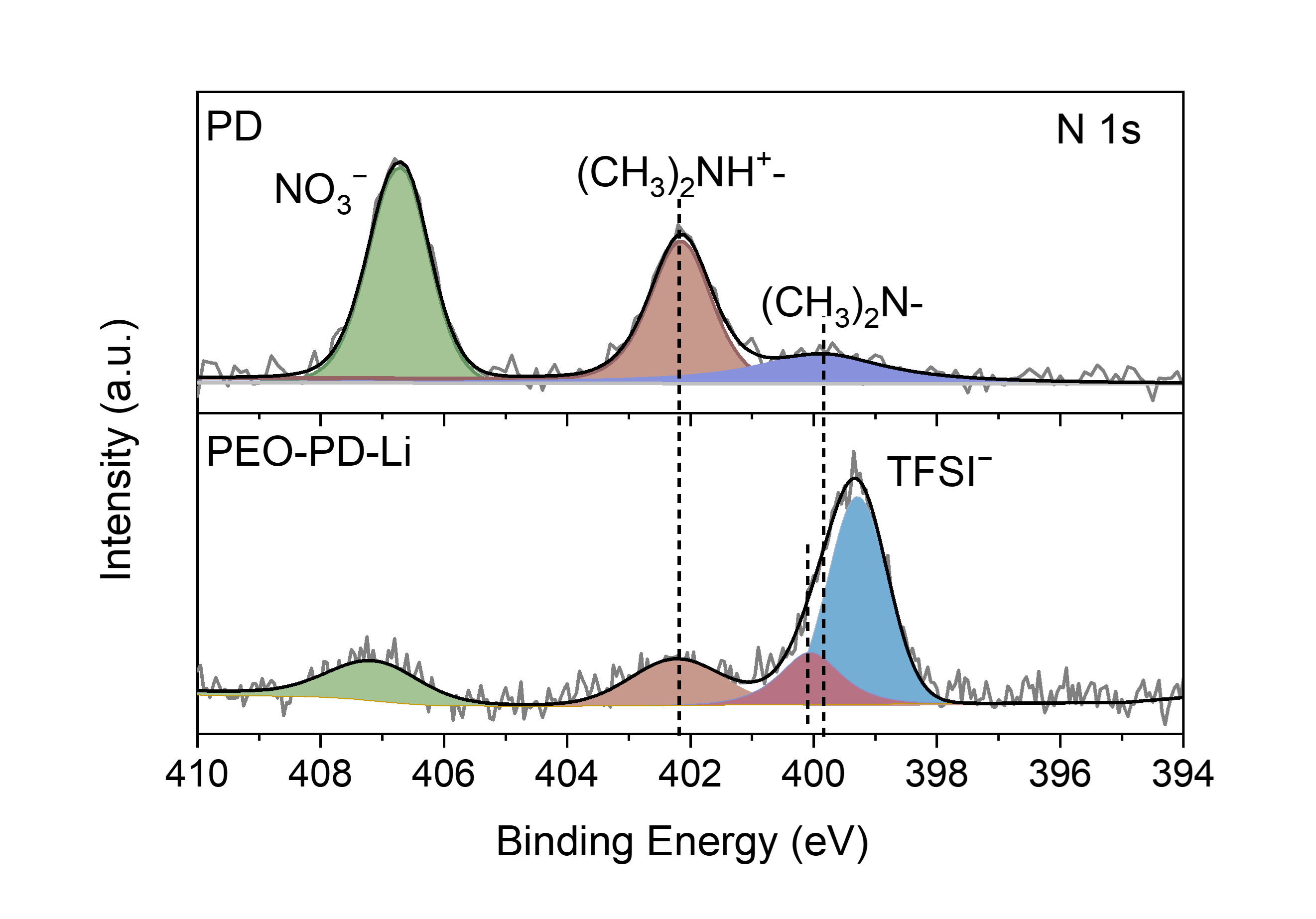


Figure S20. N 1s XPS spectra for PD powder and PEO-PD-Li electrolyte.


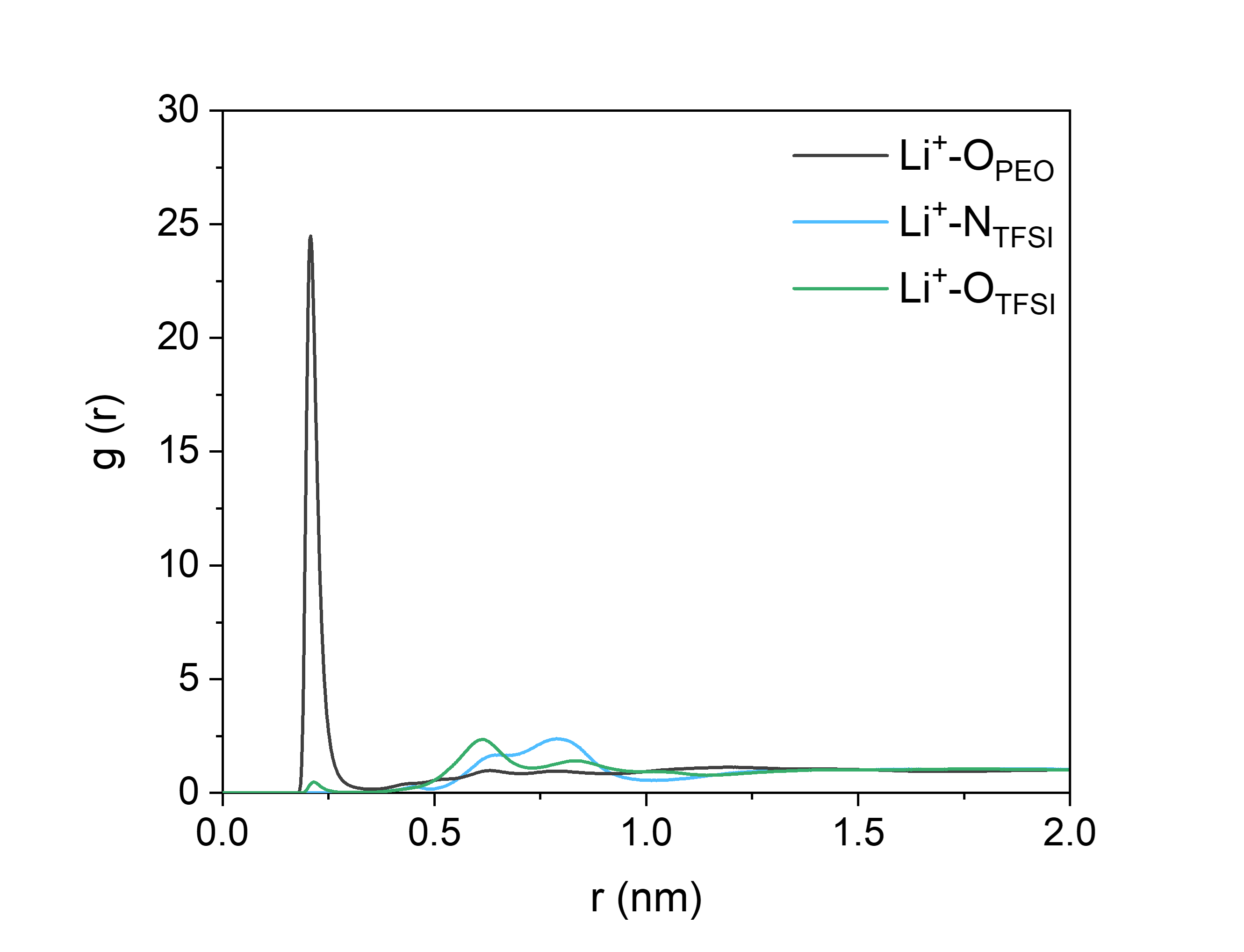


Figure S21. The Li^+^ ionic solvation structure calculated from MD simulation for the PEO-PEG-Li electrolyte.


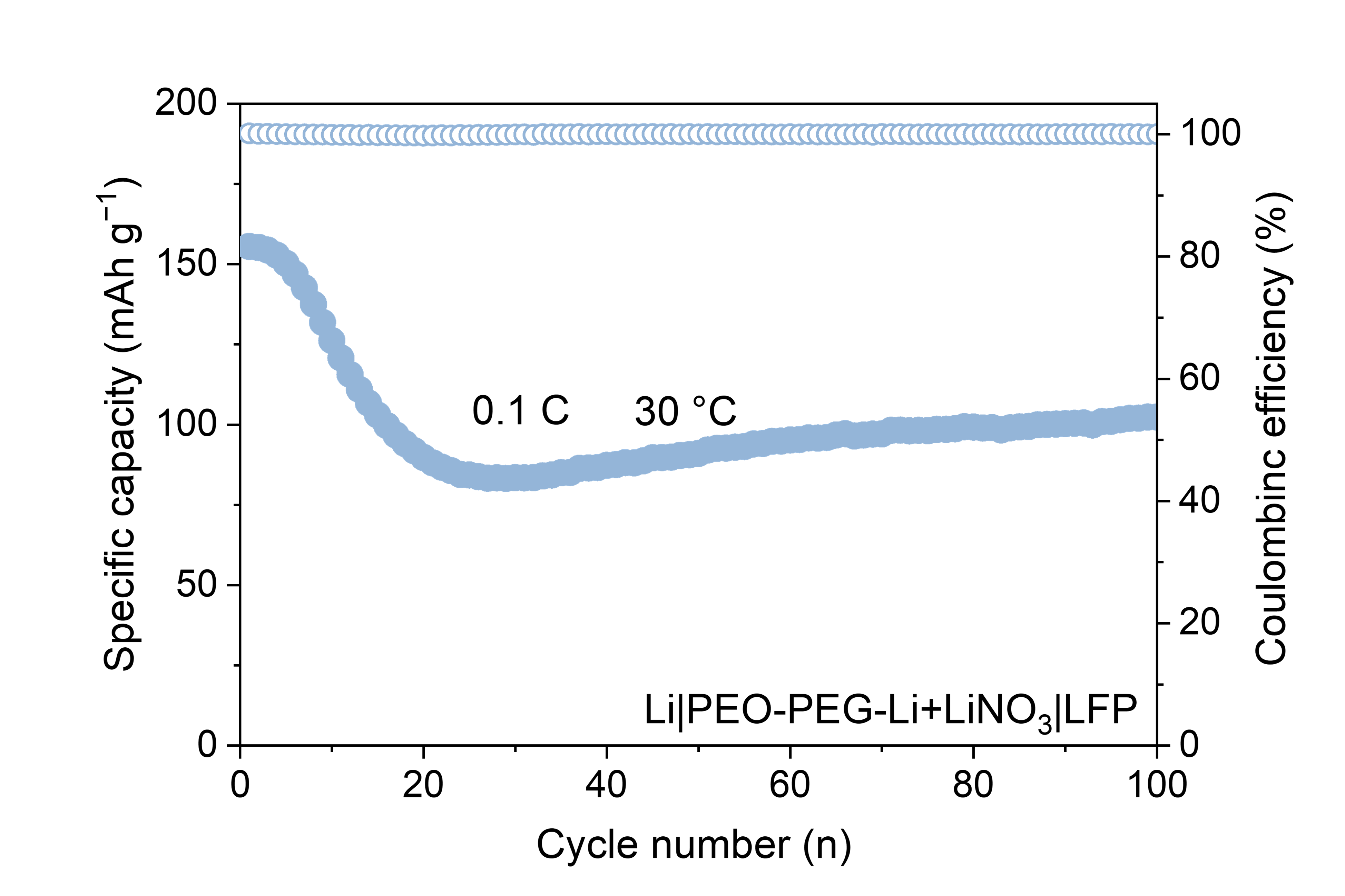


Figure S22. Cycle performance of the Li||LFP cells with PEO-PEG-Li+LiNO_3_ electrolyte at 30 °C.


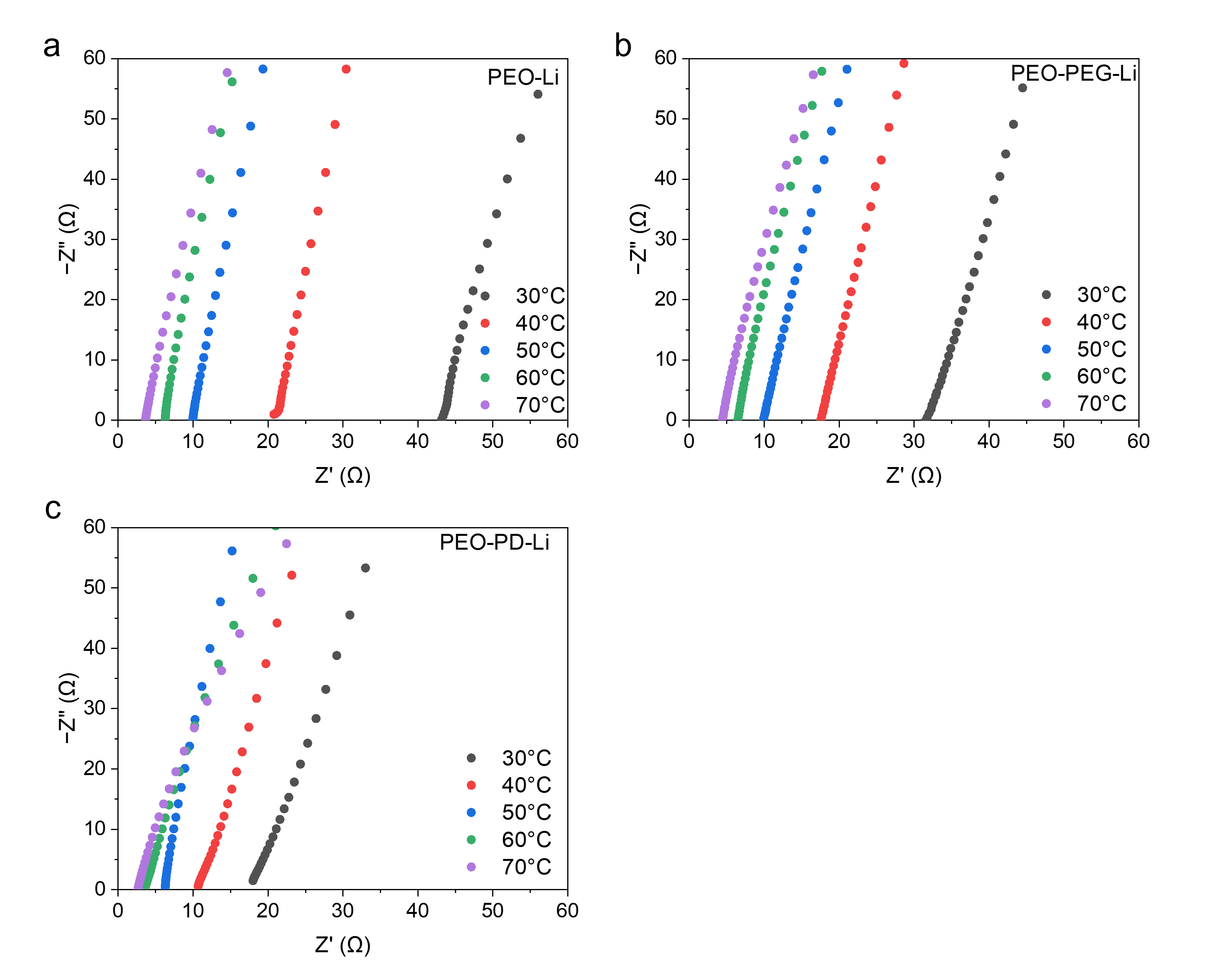


Figure S23. Electrochemical impedance spectra of the SS||SS cells assembled with different electrolytes at various temperature from 30 °C to 70 °C.


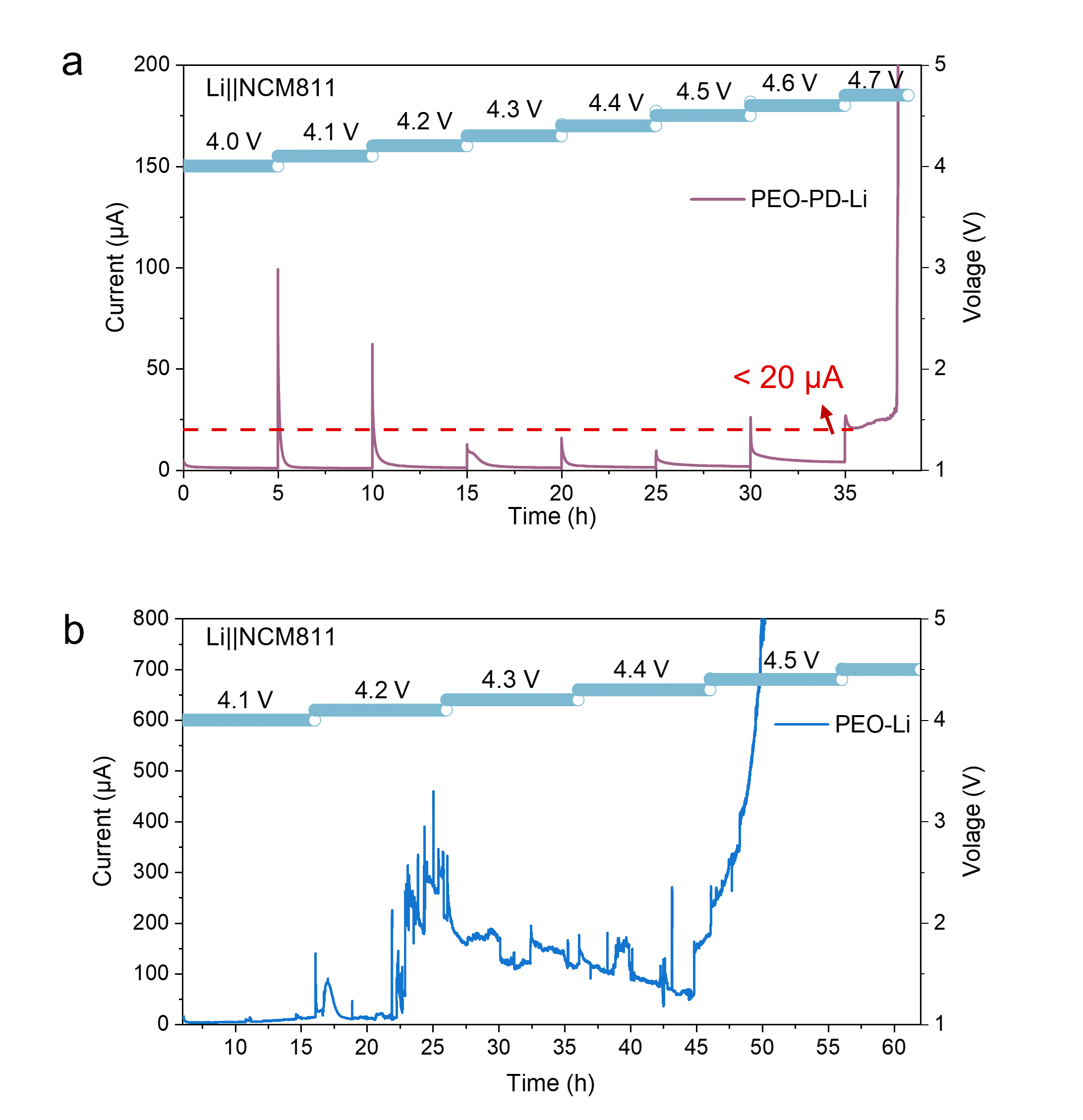


Figure S24. Electrochemical floating analyses of the (a) Li|PEO-PD-Li|NCM811 and (b) Li|PEO-Li|NCM811 cells at various voltage.


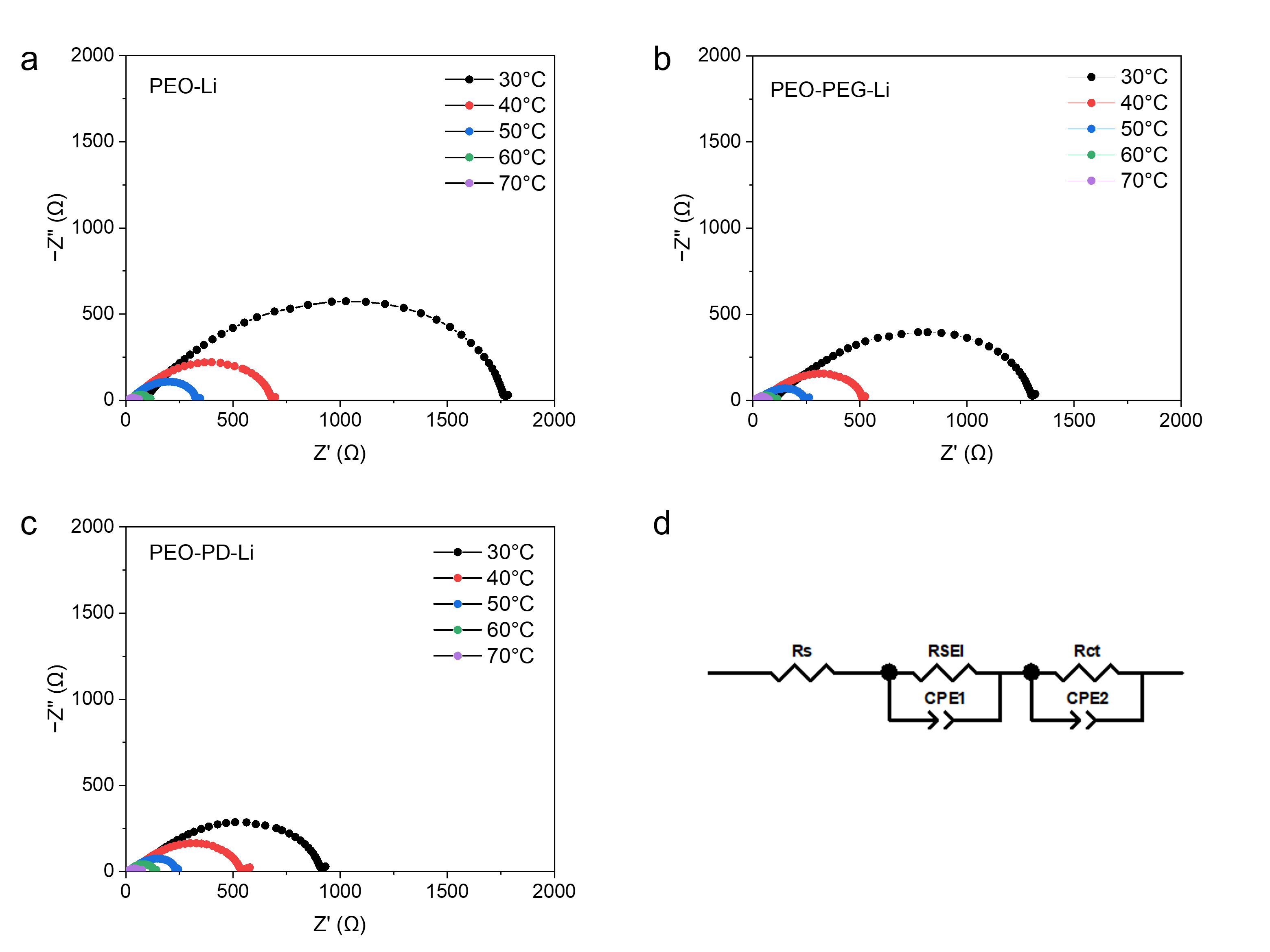


Figure S25. Electrochemical impedance spectra of the Li||Li symmetric cells assembled with (a) PEO-Li, (b) PEO-PEG-Li and (c) PEO-PD-Li electrolytes at various temperatures from 30 °C to 70 °C. (d) The equivalent circuit used for fitting the obtained profiles. (R_s_: electrolyte resistance; R_SEI_: resistance resulted from the SEI layer, which is reflected in the high-frequency impedance; R_ct_: the charge transfer resistance, related to the process of Li^+^ desolvation, which is reflected in the middle-frequency impedance.; CPE: constant phase element.)


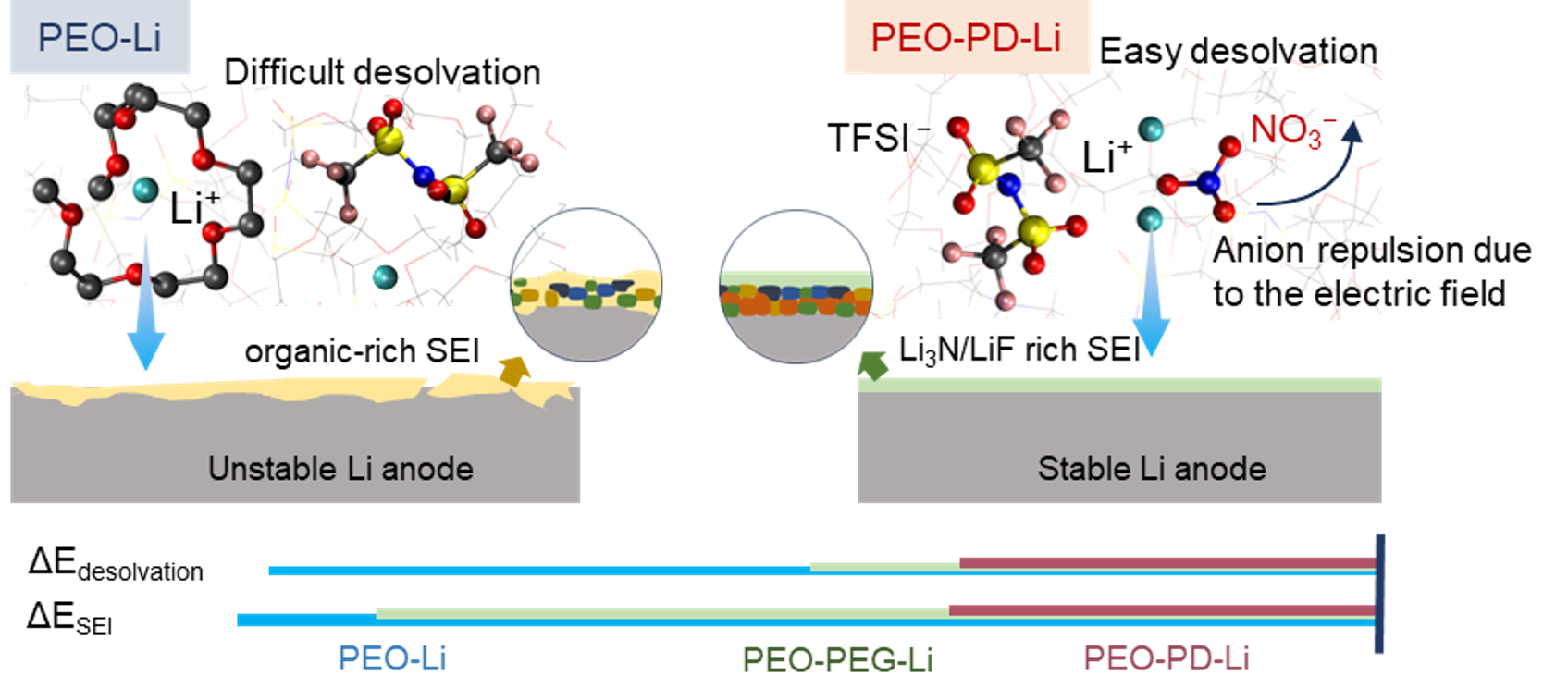


Figure S26. Schematic illustration of the improved interfacial properties for Li⁺ transport.


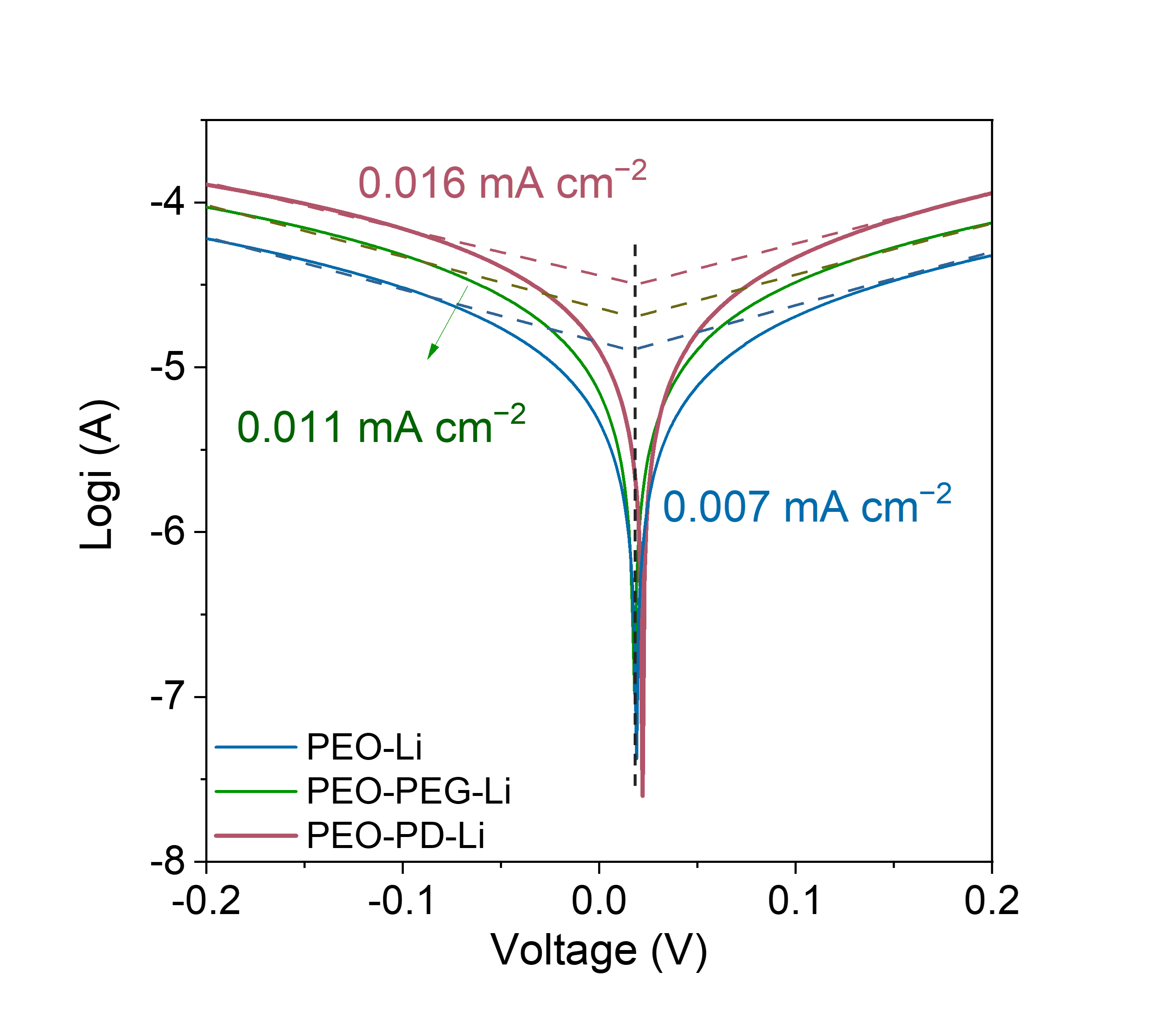


Figure S27. Tafel plots of Li||Li symmetrical cells with different electrolytes.


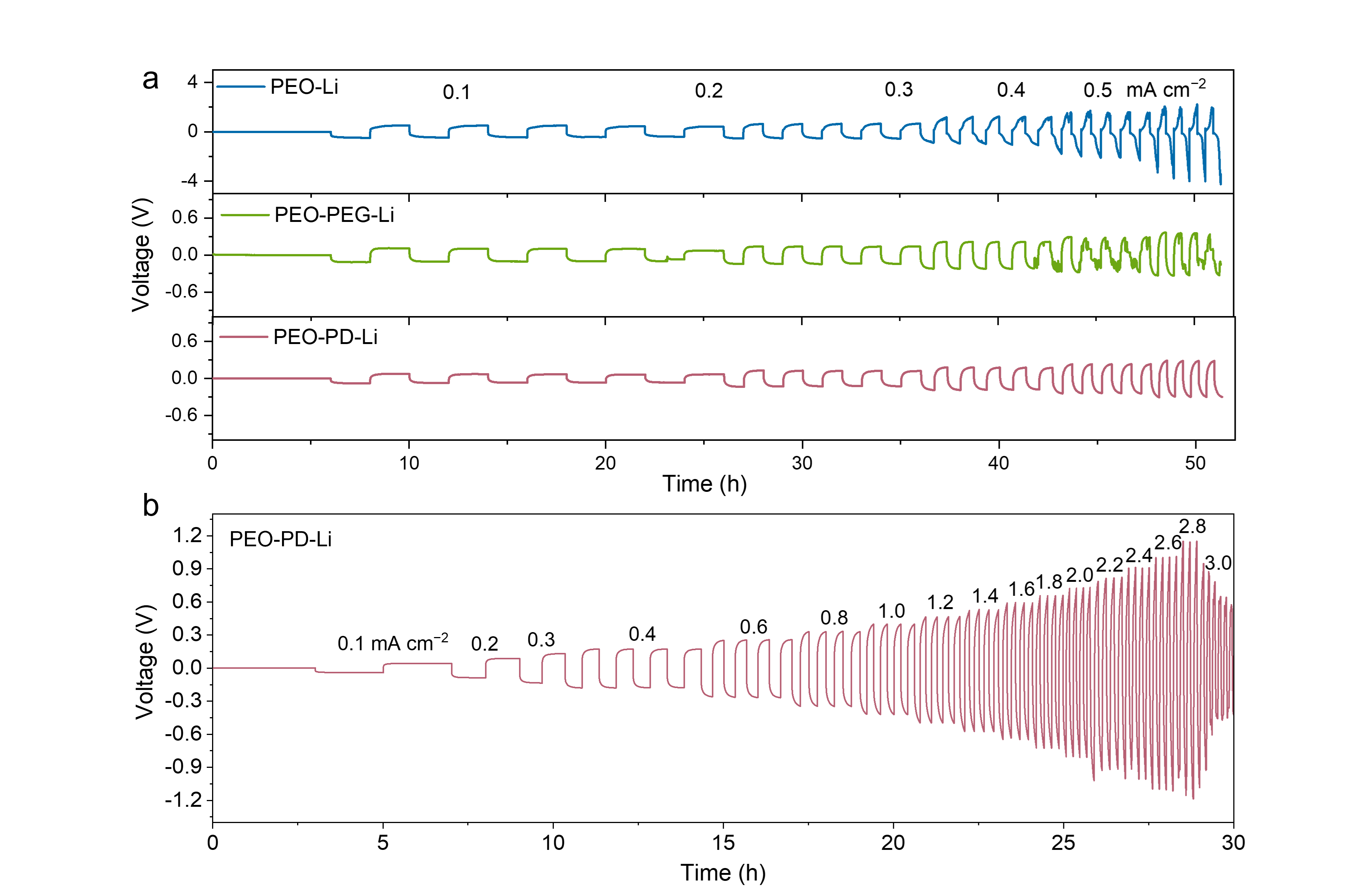


Figure S28. (a) The electrochemical performance of Li||Li symmetrical cells assembled with different electrolytes at various currents from 0.1 to 0.5 mA cm^−2^ and areal capacity of 0.2 mAh cm^−2^. (b) The electrochemical performance of Li|PEO-PD-Li|Li symmetrical cell at various currents from 0.3 to 3.0 mA cm^−2^ and areal capacity of 0.3 mAh cm^−2^.


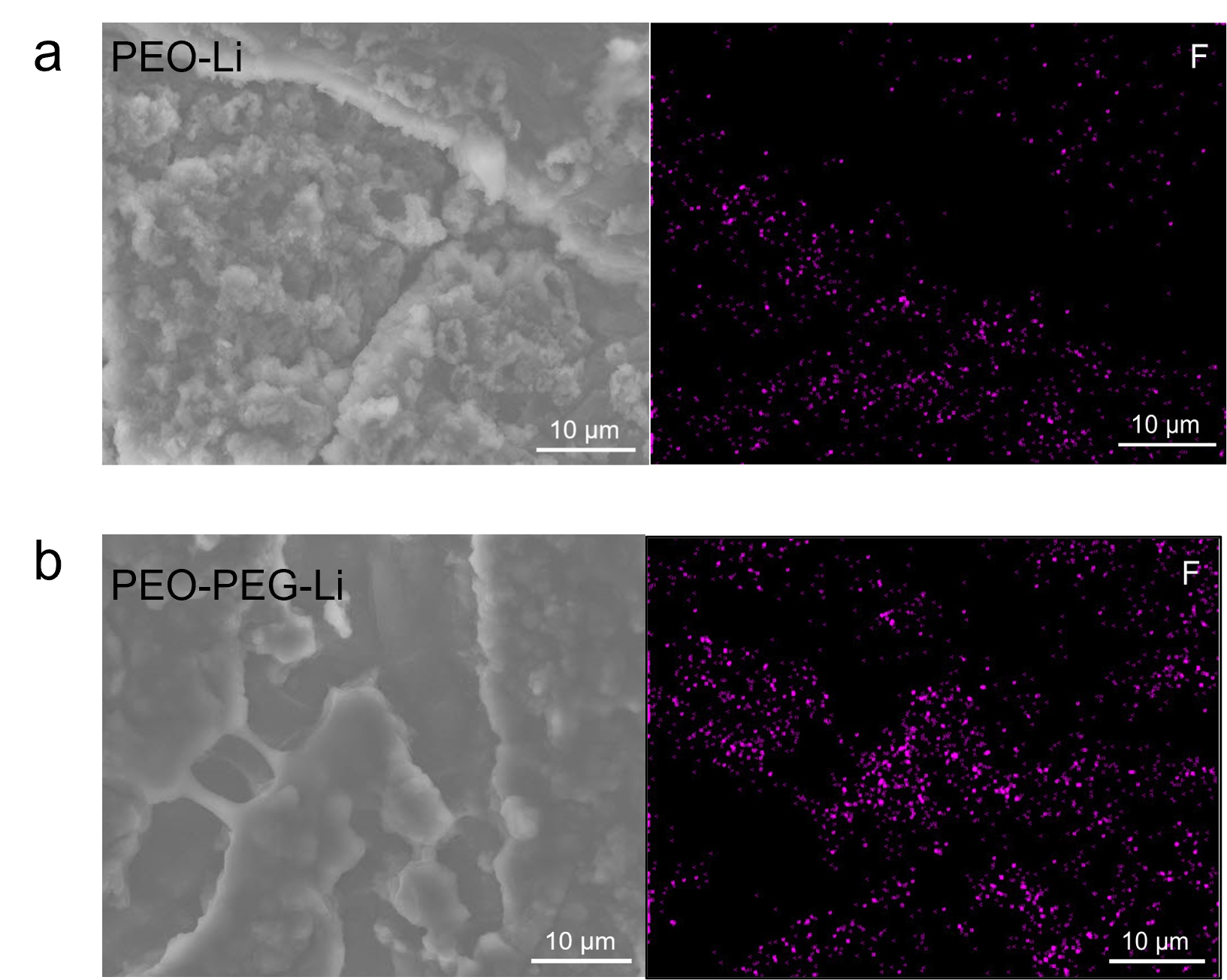


Figure S29. The SEM and EDS mapping images for F element of the cycled lithium anodes in the cells assembled with (a) PEO-Li and (b) PEO-PEG-Li electrolyte.


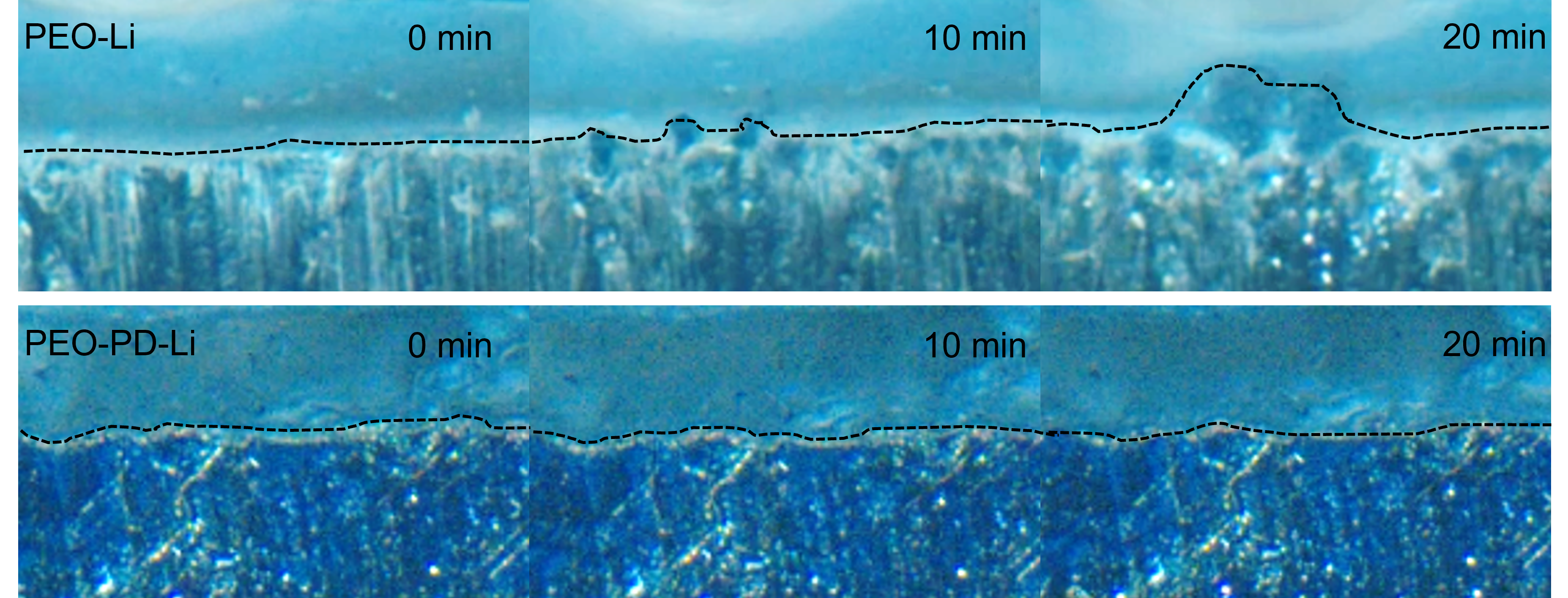


Figure S30. In operando observation of dendrite suppression at the cross-sectional of lithium symmetric cells equipped with PEO-Li and PEO-PD-Li electrolyte, respectively.


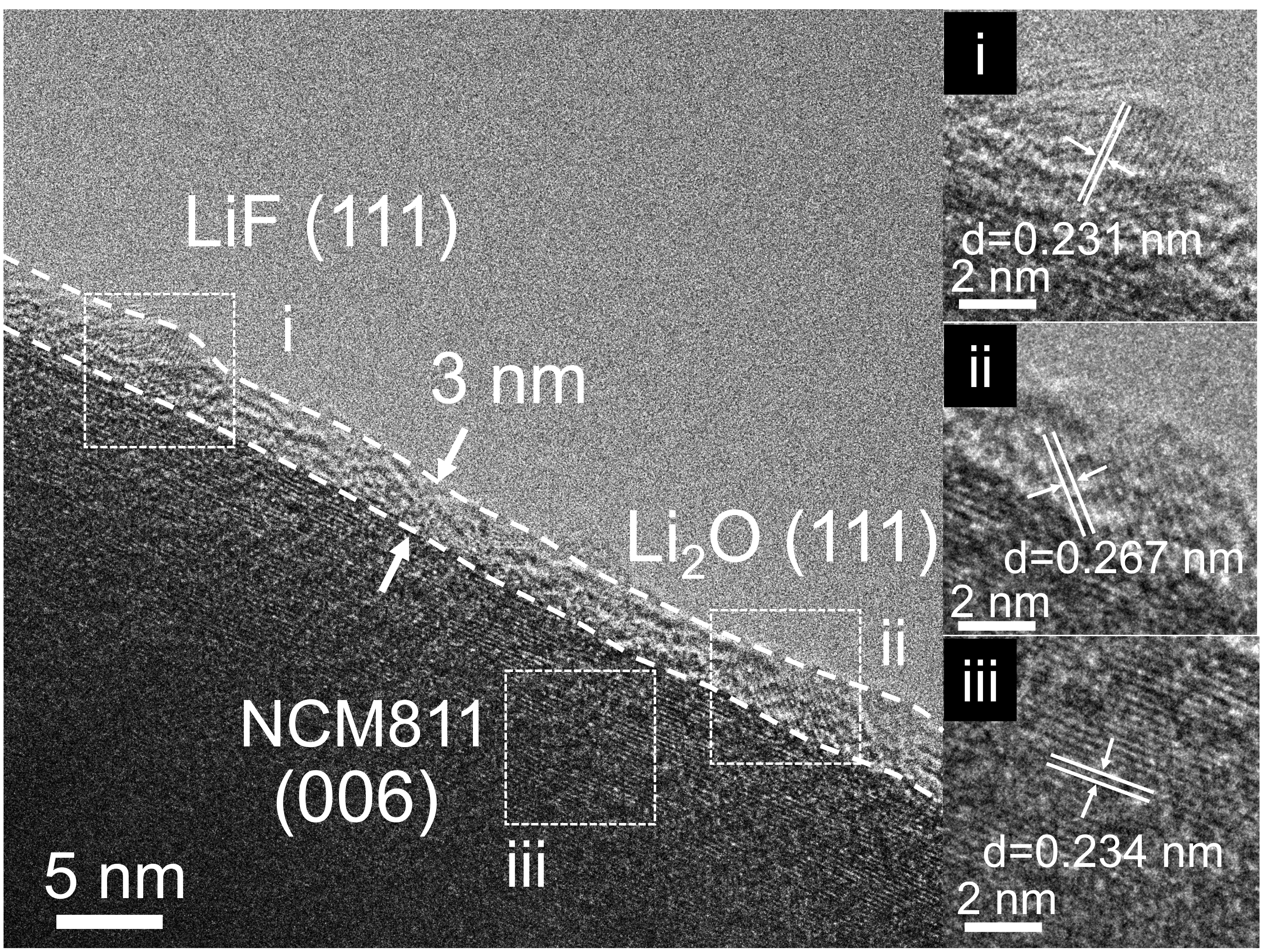


Figure S31. HRTEM image of NCM811 particle after 50 cycles in the PEO-PD-Li based cell. A high-magnification view of the boxed region is shown on the right.


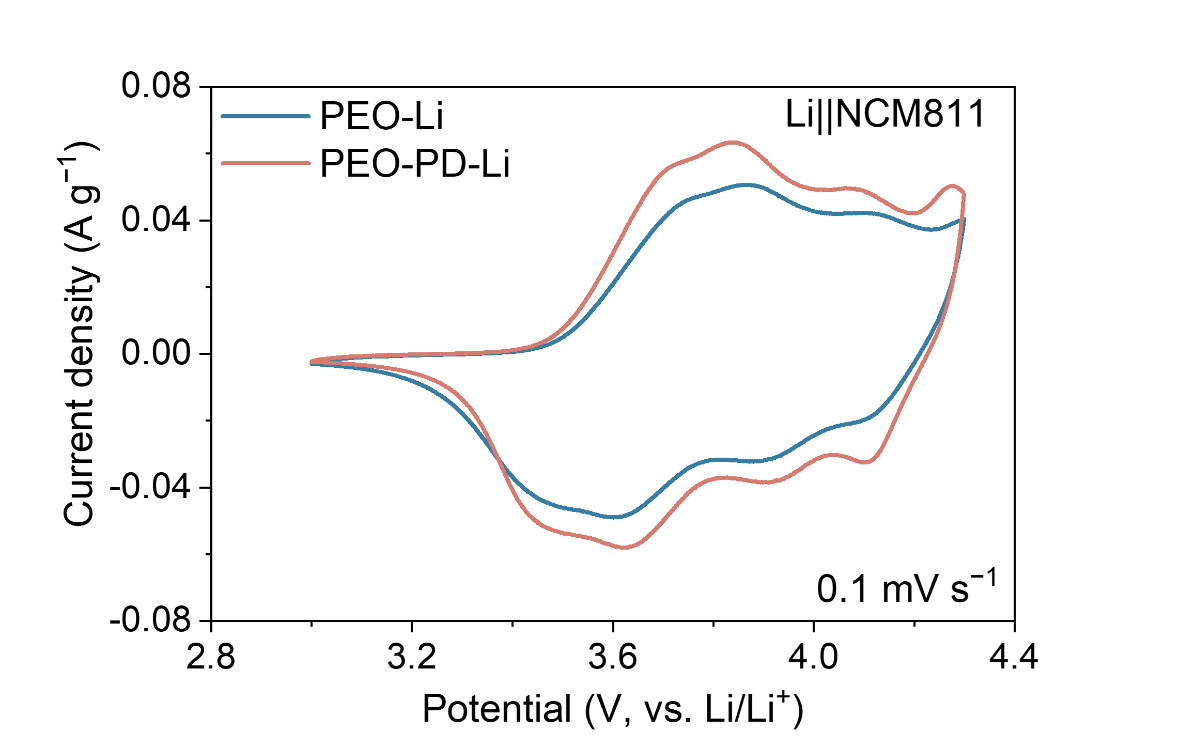


Figure S32. CV curves for Li||NCM811 cells assembled with PEO-Li and PEO-PD-Li electrolyte, respectively.


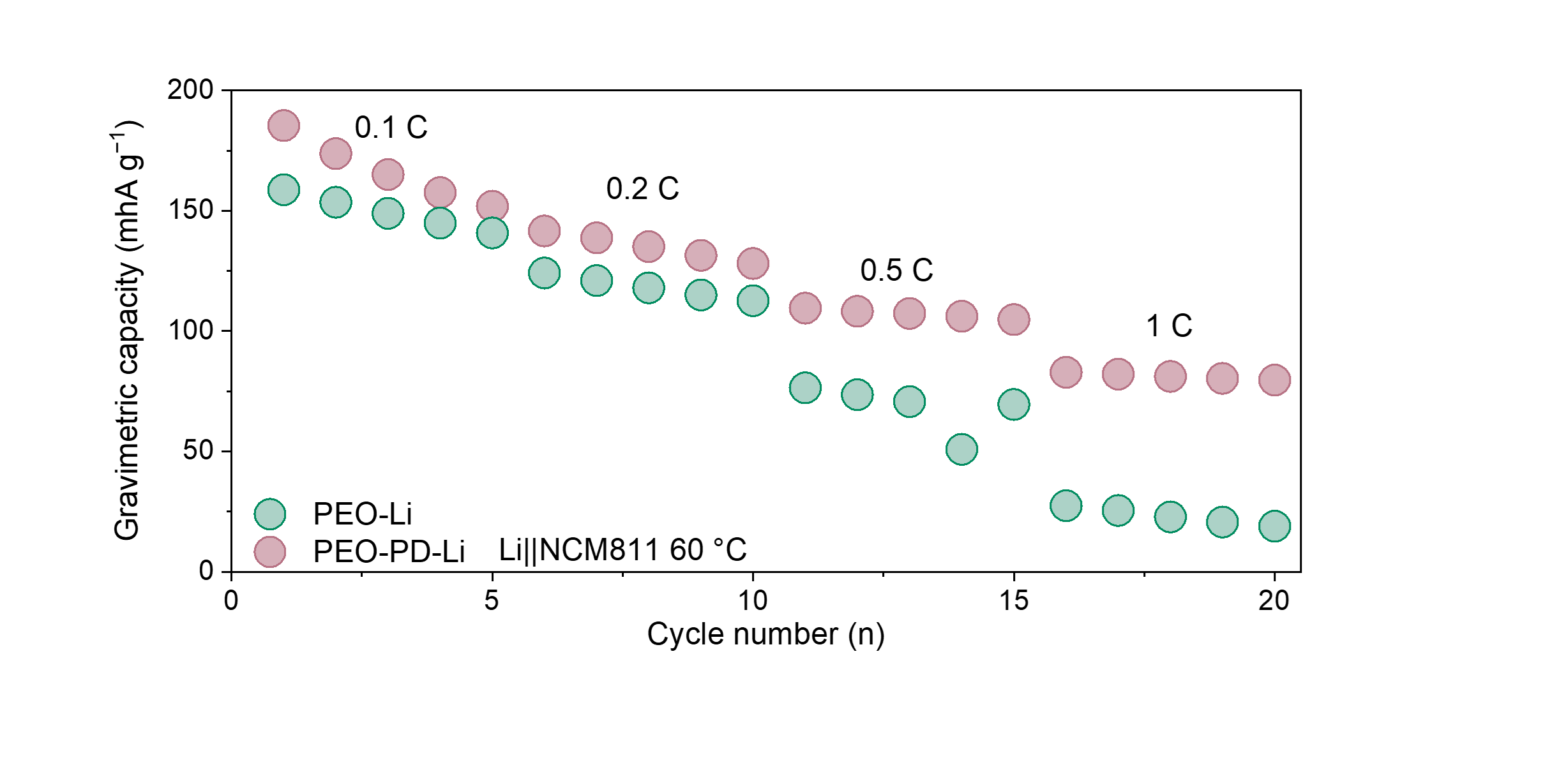


Figure S33. Rate performance of Li||NCM811 cells assembled with PEO-Li and PEO-PD-Li electrolytes at 60 °C.


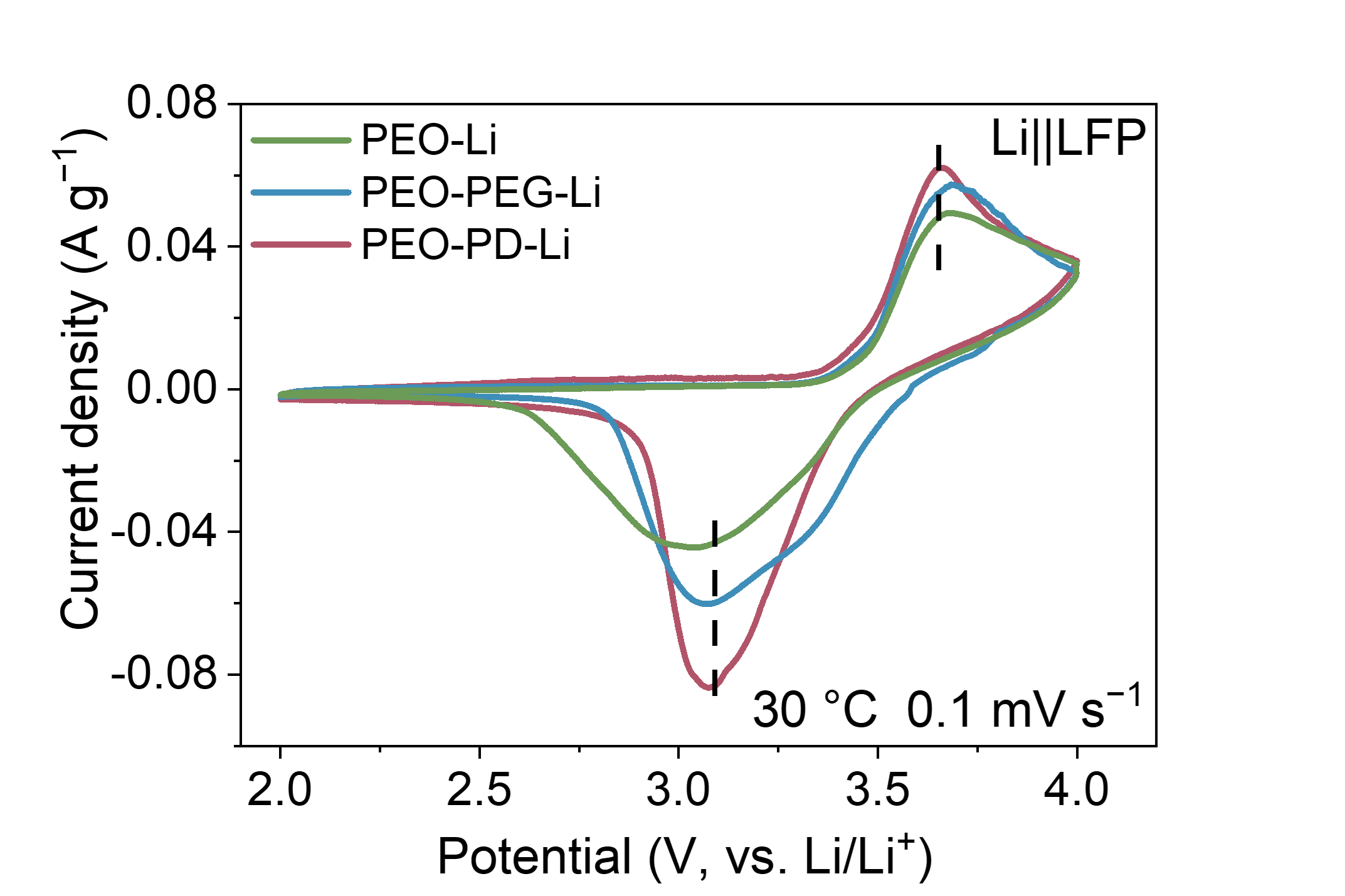


Figure S34. CV curves for Li||LFP cells assembled with different electrolytes.


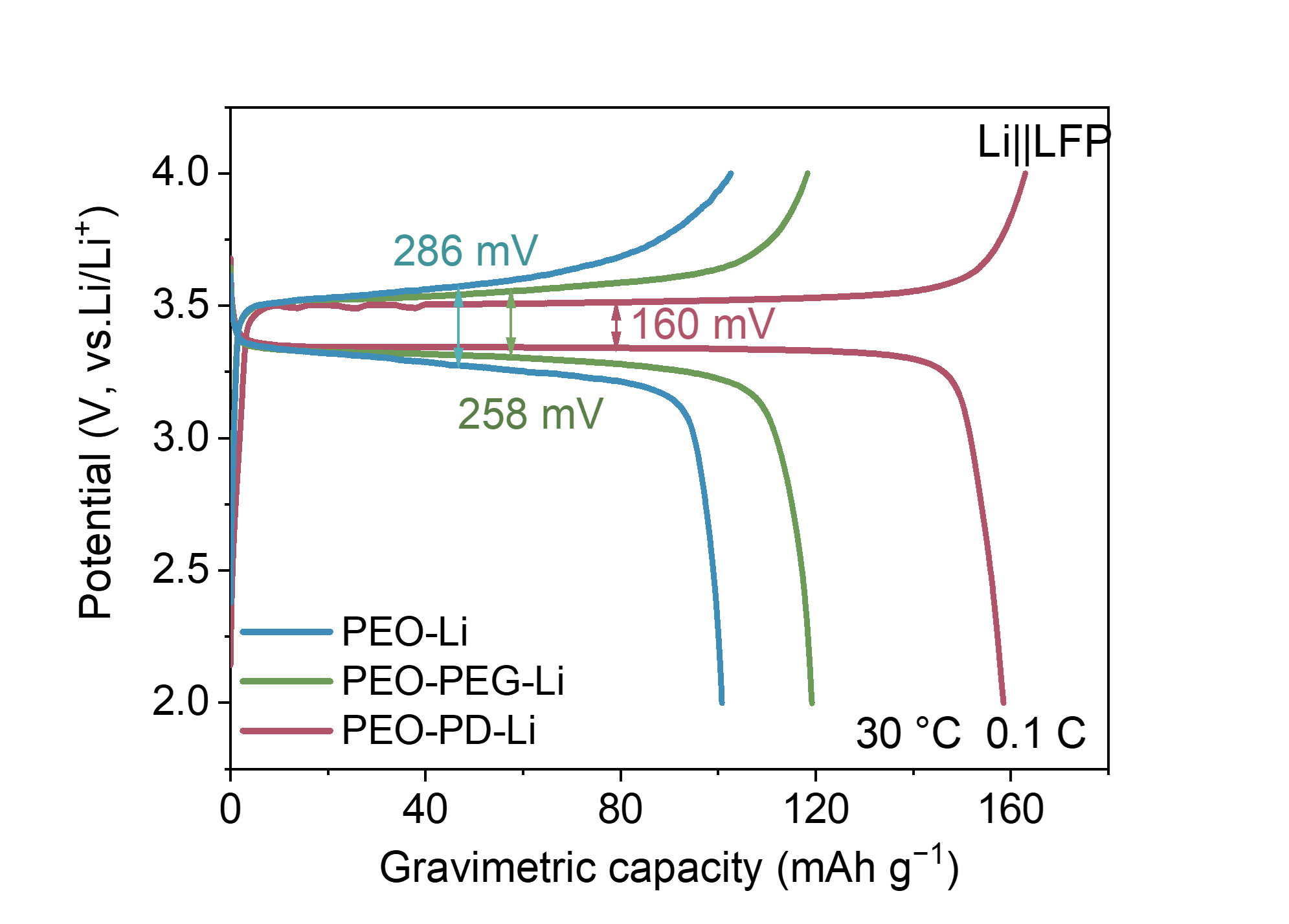


Figure S35. Charge/discharge curves at 0.1 C for Li||LFP cells assembled with different electrolytes.


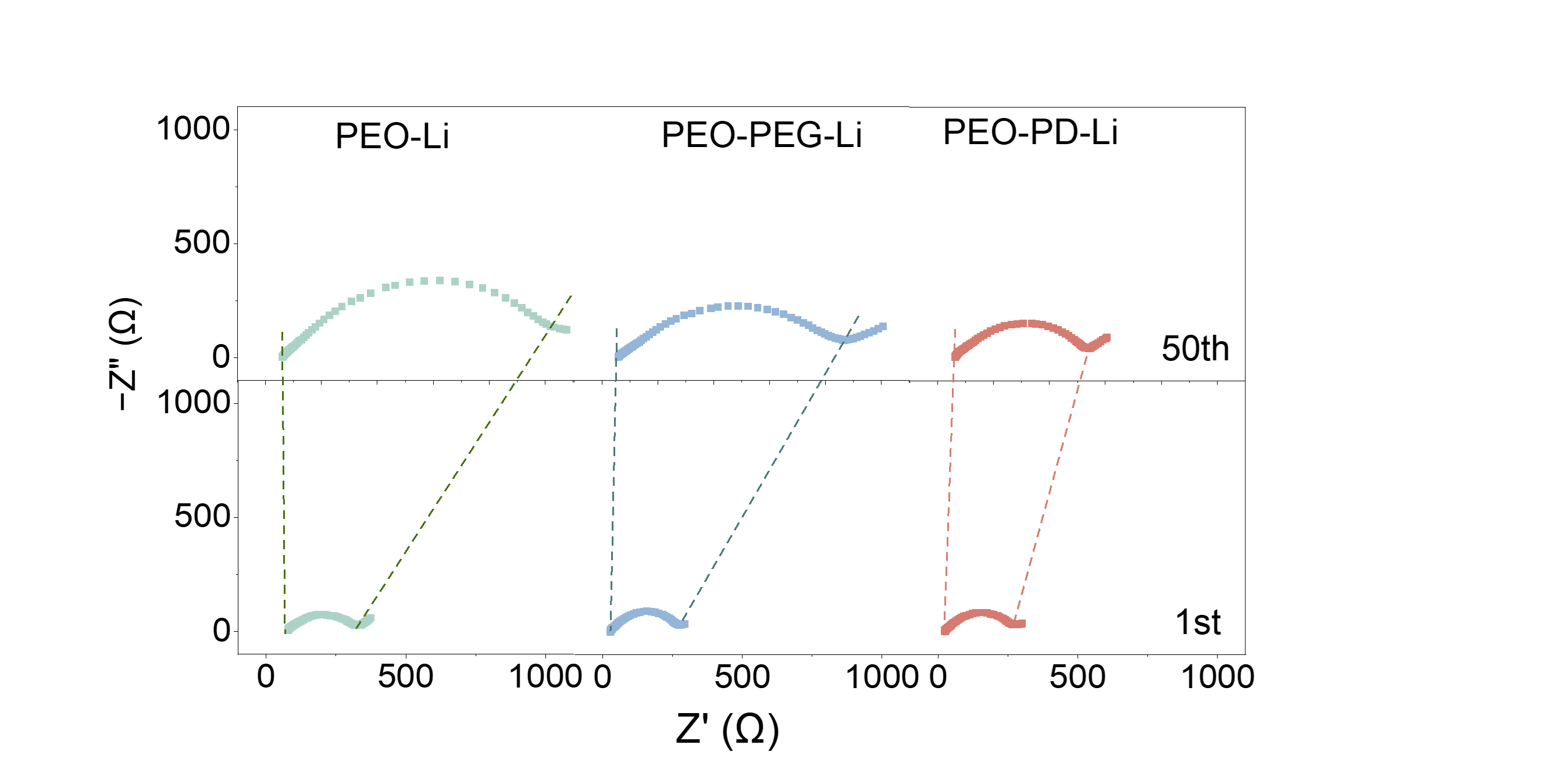


Figure S36. EIS plots of the Li||LFP cells assembled with PEO-Li, PEO-PEG-Li and PEO-PD-Li electrolytes at the 1st and 50th cycles.


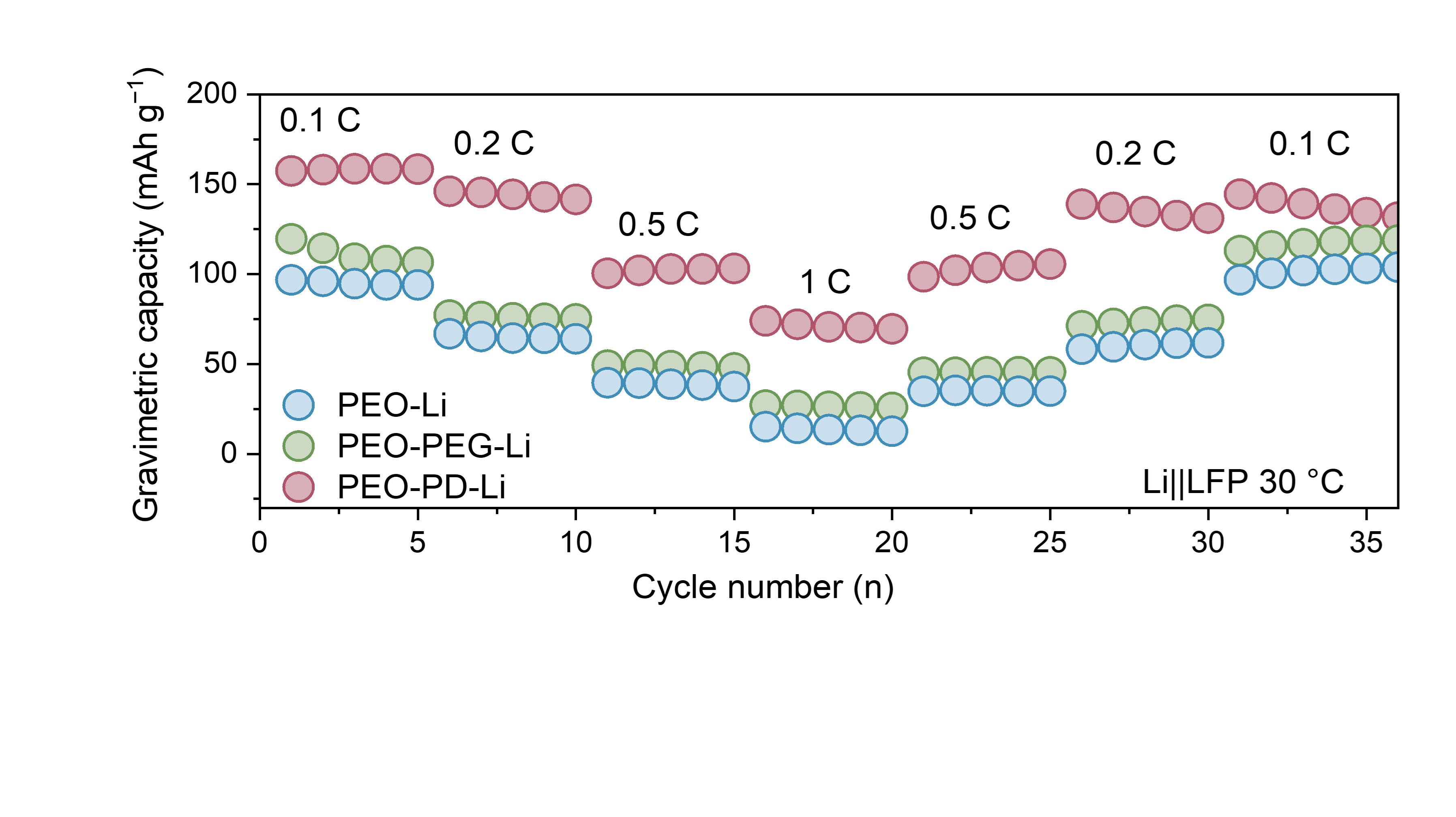


Figure S37. Rate performance of Li||LFP cells assembled with different electrolytes.


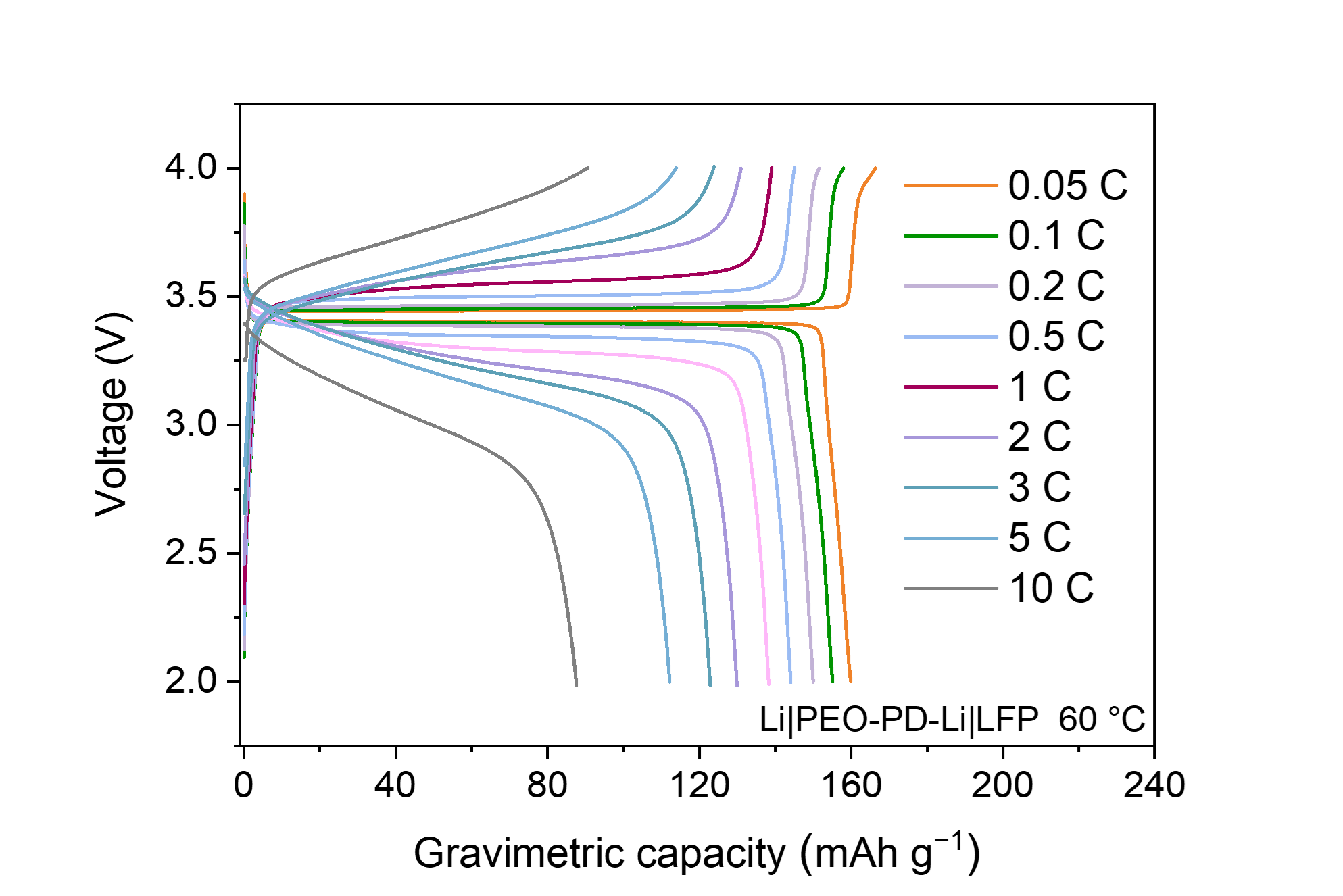


Figure S38. Charge/discharge curves of Li|PEO-PD-Li|LFP cell at different currents


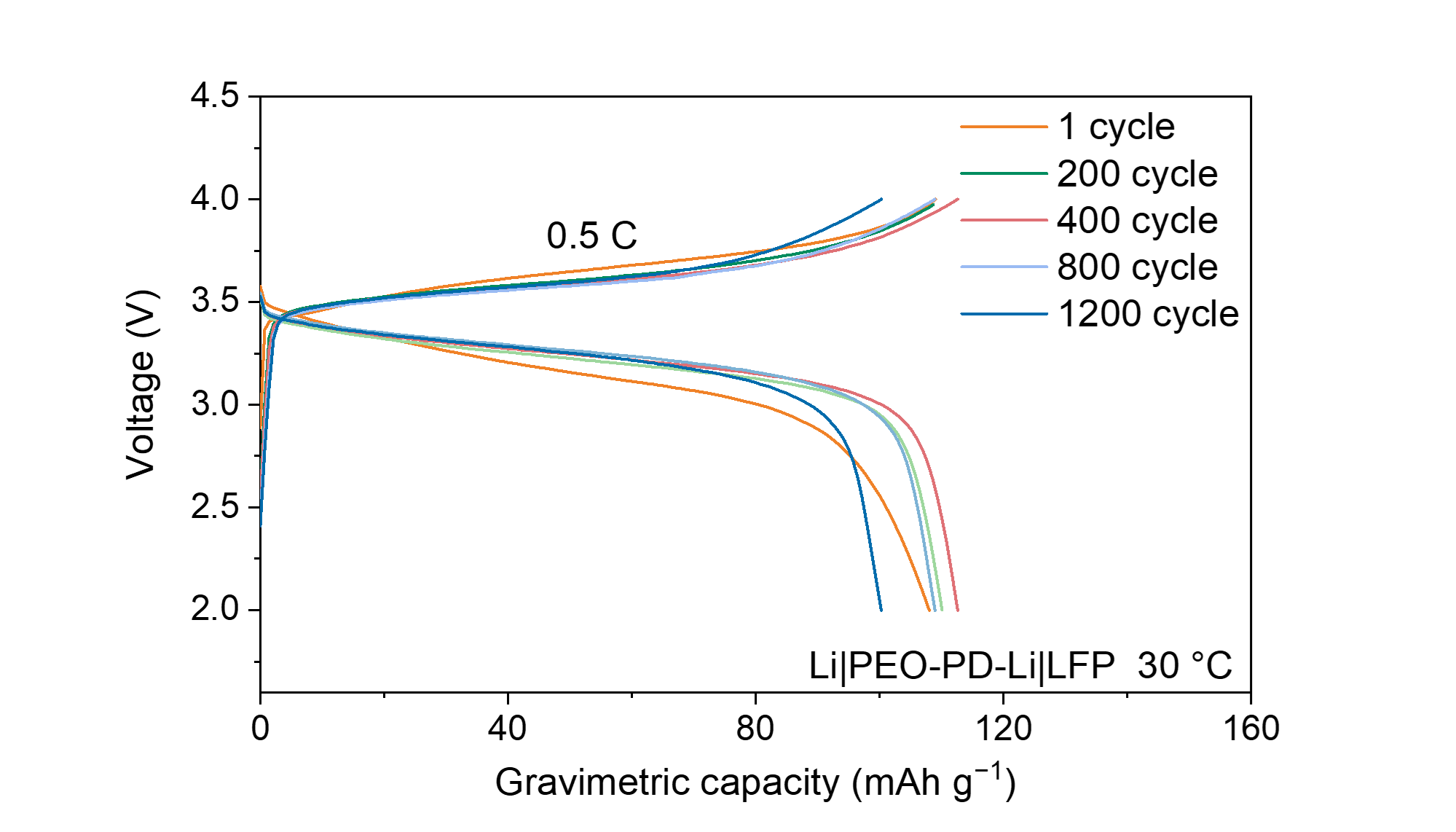


Figure S39. Charge/discharge curves at different cycles of Li||LFP cell assembled with PEO-PD-Li electrolyte at 0.5 C.


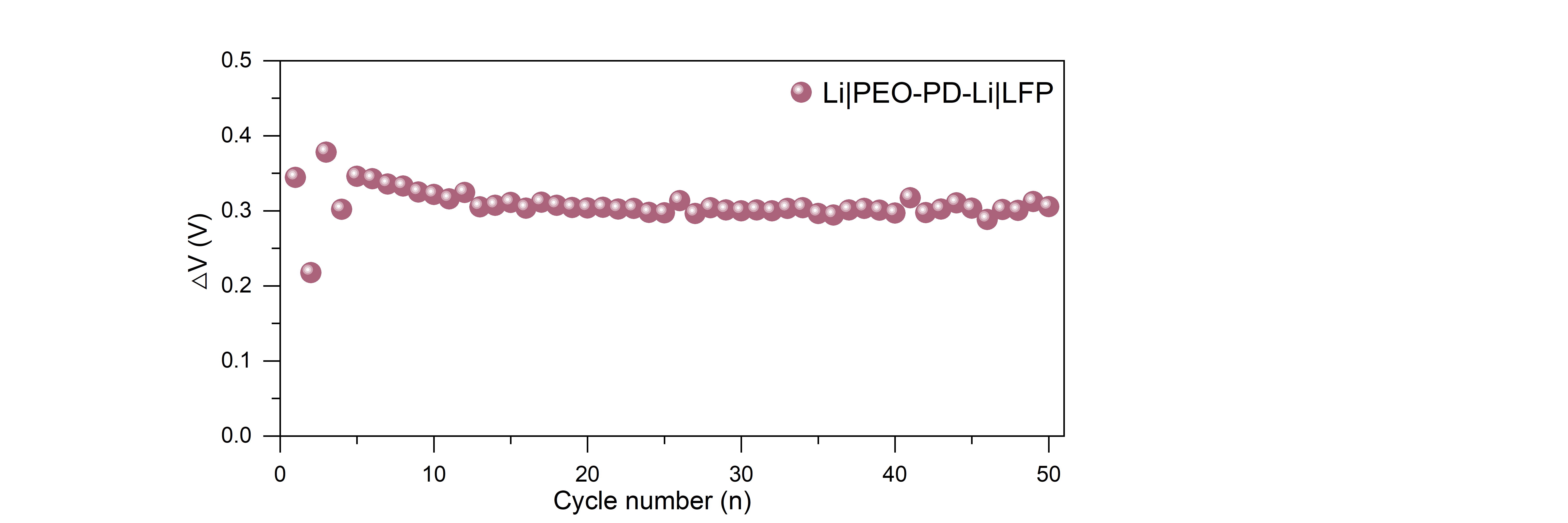


Figure S40. The polarization voltage of the Li|PEO-PD-Li|LFP pouch cell during the cycling at 0.1 C and 35 °C.

# Supporting Tables

**Table S1.** Molecular counts of the constituent species in the MD simulations of the three electrolytes.

| System Name | PEO_100_^(a)^ | LiTFSI | PEG_20_^(b)^ | PD^+^ NO_3_^− (c)^ |
| --- | --- | --- | --- | --- |
| PEO-Li | 50 | 500 | 0 | 0 |
| PEO-PEG-Li | 30 | 500 | 100 | 0 |
| PEO-PD-Li | 30 | 500 | 0 | 100 |

(a) PEO_100_: poly(ethylene oxide) chains with 100 repeat EO (-CH_2_CH_2_O-) units; (b) PEG_20_: poly(ethylene glycol) additive with 20 repeat EO units; (c) PD^+^ NO_3_^−^: additive consisting of 18 repeat EO units terminated with two DMAEMA chains, one bearing tertiary amine group, the other a quaternary ammonium group, associated with a NO_3_^−^ counterion. The molecular structures of all components are illustrated in Figure S16.

**Table S2.** Fitted date for the EIS curves of Li||Li symmetric cells assembled with PEO-Li, PEO-PEG-Li and PEO-PD-Li electrolytes at various temperatures.

| Temperature  (K) | R*_SEI_* (Ω) | | | R*_ct_* (Ω) | | |
| --- | --- | --- | --- | --- | --- | --- |
|  | PEO-Li | PEO-PEG-Li | PEO-PD-Li | PEO-Li | PEO-PEG-Li | PEO-PD-Li |
| 303 | 467 | 335 | 189 | 1227 | 887 | 665 |
| 313 | 135 | 150 | 100 | 500 | 419 | 382 |
| 323 | 48 | 45 | 41 | 264 | 180 | 180 |
| 333 | 17 | 17 | 19 | 80 | 73 | 90 |
| 343 | 12 | 11 | 12 | 29 | 48 | 39 |

# References

[1] L. Bromberg, S. Deshmukh, M. Temchenko, L. Iourtchenko, V. Alakhov, C. Alvarez-Lorenzo, R. Barreiro-Iglesias, A. Concheiro, *Bioconjugate Chem*. **2005**, *16*, 626-633.

[2] A. Du, H. Lu, S. Liu, S. Chen, Z. Chen, W. Li, J. Song, Q. H. Yang, *Adv. Energy Mater.* **2024**, *14*, 2400808.

[3] G.Y. Gu, S. Bouvier, C. Wu, R. Laura, M. Rzeznik, K.M. Abraham, *Electrochim*. *Acta* **2000**, *45*, 3127-3139.

[4] K.M. Diederichsen, H.G. Buss, B.D. McCloskey, *Macromolecules* **2017**, *50*, 3831-3840.

[5] D.G. Mackanic, X. Yan, Q. Zhang, N. Matsuhisa, Z. Yu, Y. Jiang, T. Manika, J. Lopez, H.Yan, K. Liu, X. Chen, Y. Cui, Z. Bao, *Nat*. *Commun*. **2019**, *10*, 5384.

[6] M. J. Abraham, T. Murtola, R. Schulz, S. Páll, J. C. Smith, B. Hess, E. Lindahl, *SoftwareX,*  **2015**, *1*, 19-25.

[7] L. S. Dodda, I. Cabeza de Vaca, J. Tirado-Rives, W. L. Jorgensen, *Nucleic Acids Res.* **2017**, *45*, W331-W336.

[8] B. Doherty, X. Zhong, S. Gathiaka, B. Li, O. Acevedo, *J. Chem. Theory Comput*. **2017**, *13*, 6131-6145.

[9] T. Lu, F. J. Chen, *J. Comput. Chem.* **2012**, *33*, 580-592.

[10] M. Frisch, G. Trucks, H. Schlegel, G. Scuseria, M. Robb, J. Cheeseman, G. Scalmani, V. Barone, G. Petersson, H. J. W. Nakatsuji, CT, **2016**.

[11] aA. D. J. T. J. o. C. P. d. h. d. o. Becke, **1993**; bS. H. Vosko, L. Wilk, M. Nusair, *Can. J. Phys.* **1980**, *58*, 1200-1211; cP. J. Stephens, F. J. Devlin, C. F. Chabalowski, M. J. Frisch, *J. Phys. Chem.* **1994**, *98*, 11623-11627.

[12] S. Grimme, J. Antony, S. Ehrlich, H. Krieg, *J. Chem. Phys.,***2010**, *132*, 154104.

[13] S. Grimme, S. Ehrlich, L. Goerigk, *J. Comput. Chem,* **2011**, *32*, 1456-1465.

[14] S. Grimme, *Chem. Eur. J*. **2012**, *18*, 9955-9964.

[15] J. P. Merrick, D. Moran, L. Radom, *J. Phys. Chem. A* , **2007**, *111*, 11683-11700.

[16] S. Miertuš, E. Scrocco, J. Tomasi, *Chem. Phys*, **1981**, *55*, 117-129.

[17] K. Se, K. Adachi, T. Kotaka, *Polym. J*, **1981**, *13*, 1009-1017.
